# A partial reduction of VDAC1 enhances mitophagy, autophagy, synaptic activities in a transgenic Tau mouse model

**DOI:** 10.1111/acel.13663

**Published:** 2022-07-07

**Authors:** Murali Vijayan, Rainier Vladlen Alvir, Razelle Veronique Alvir, Lloyd E. Bunquin, Jangampalli Adi Pradeepkiran, P. Hemachandra Reddy

**Affiliations:** ^1^ Department of Internal Medicine Texas Tech University Health Sciences Center Lubbock Texas USA; ^2^ Department of Pharmacology and Neuroscience Texas Tech University Health Sciences Center Lubbock Texas USA; ^3^ Department of Neurology Texas Tech University Health Sciences Center Lubbock Texas USA; ^4^ Department of Public Health, Graduate School of Biomedical Sciences Texas Tech University Health Sciences Center Lubbock Texas USA; ^5^ Department of Speech, Language, and Hearing Sciences Texas Tech University Health Sciences Center Lubbock Texas USA

**Keywords:** Alzheimer's disease, autophagy, hexokinases, mitochondria, mitochondrial biogenesis, mitophagy, oxidative stress, voltage‐dependent anion channel 1

## Abstract

Alzheimer's disease (AD) is the most common cause of mental dementia in the aged population. AD is characterized by the progressive decline of memory and multiple cognitive functions, and changes in behavior and personality. Recent research has revealed age‐dependent increased levels of VDAC1 in postmortem AD brains and cerebral cortices of APP, APPxPS1, and 3xAD.Tg mice. Further, we found abnormal interaction between VDAC1 and P‐Tau in the AD brains, leading to mitochondrial structural and functional defects. Our current study aimed to understand the impact of a partial reduction of voltage‐dependent anion channel 1 (VDAC1) protein on mitophagy/autophagy, mitochondrial and synaptic activities, and behavior changes in transgenic TAU mice in Alzheimer's disease. To determine if a partial reduction of VDAC1 reduces mitochondrial and synaptic toxicities in transgenic Tau (P301L) mice, we crossed heterozygote VDAC1 knockout (VDAC1^+/−^) mice with TAU mice and generated double mutant (VDAC1^+/−^/TAU) mice. We assessed phenotypic behavior, protein levels of mitophagy, autophagy, synaptic, other key proteins, mitochondrial morphology, and dendritic spines in TAU mice relative to double mutant mice. Partial reduction of VDAC1 rescued the TAU‐induced behavioral impairments such as motor coordination and exploratory behavioral changes, and learning and spatial memory impairments in VDAC1^+/−^/TAU mice. Protein levels of mitophagy, autophagy, and synaptic proteins were significantly increased in double mutant mice compared with TAU mice. In addition, dendritic spines were significantly increased; the mitochondrial number was significantly reduced, and mitochondrial length was increased in double mutant mice. Based on these observations, we conclude that reduced VDAC1 is beneficial in symptomatic‐transgenic TAU mice.

## Graphical abstract

Schematic overview of VDAC1^+/+^ and VDAC1^+/−^ model: Working model for the regulation of mitophagy, mitochondrial dynamics, mitochondrial biogenesis, VDAC1, and Hexokinase interaction, and VDAC1 and P‐Tau interaction. In VDAC1^+/+^, (i) dissociation of hexokinase (HK) results in a “closed” VDAC configuration that prevents adenine nucleotide exchange and uncouples intra and extramitochondrial metabolism, (ii) shifts the equilibrium to the VDAC1 oligomeric state, mediating the release of apoptogenic proteins and leading to apoptosis, (iii) Elevated GSK3β activity is a key event in phosphorylation of Tau and synaptic pathology in AD. GSK3β is proposed to activate VDAC1 phosphorylation, ultimately leading to mitochondrial dysfunction and synaptic damage in AD, (iv) Defective mitophagy, (v) increased mitochondrial fission and decreased mitochondrial fusion. In VDAC1^+/−^ (i) VDAC1 and ANT reportedly interact directly when complexed with HK, an arrangement that facilitates mitochondrial contact site formation and metabolite exchange, (ii) decreased GSK3β and P‐Tau activity, and (iii) PINK1 recruits PARKIN from the cytoplasm to mitochondrial outer membrane. After PARKIN has been recruited to mitochondria, activated PARKIN ubiquitinates proteins in the mitochondrial outer membrane VDAC to facilitate mitophagy. PARKIN‐mediated ubiquitination recruits autophagy adapter protein p62, and these proteins interact with LC3B. LC3B participates in the formation of an autophagosome, which is fated for lysosomal destruction to clear out damaged mitochondrion, (iv) decreased mitochondrial fission and increased mitochondrial fusion.

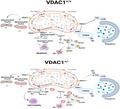

**Abbreviations**
AD: Alzheimer's disease
AKT: Serine/Threonine Kinase
ALS: Amyotrophic lateral sclerosis
APP: Amyloid precursor protein
ATG5: Autophagy Protein 5
ATP: Adenosine triphosphate
Aβ: Amyloid‐beta
CypD: Cyclophilin D
GSK: Glycogen synthase kinase
GTPase: guanosine triphosphate
H_2_O_2_
: Hydrogen Peroxide
HK: Hexokinase
LC3B: Microtubule Associated Protein 1 Light Chain 3 Beta
MFN: Mitofusins
MWM: Morris water maze
NFT: neurofibrillary tangle
PARKIN: E3 ubiquitin‐protein ligase
PINK1: Serine/threonine‐protein kinase
SEM: Standard error of the mean
SQSTM1: p62/sequestosome1
TEM: Transmission Electron Microscopy
VDAC: Voltage‐dependent anion channel
WT: Wild Type mice

## 1 INTRODUCTION

Alzheimer's disease (AD) is a late‐onset, neurodegenerative disease characterized by a progressive decline of memory and cognitive functions and changes in behavior and personality. AD results in the irreversible loss of neurons, particularly in the learning and memory regions of the brain (LaFerla et al., 2007; Selkoe, 2001). AD currently affects over 6 million people in the USA, with estimates suggesting this number will nearly triple by 2060 (Matthews et al., 2019; Vijayan et al., 2017; Vijayan & Reddy, 2016). Intracellular phosphorylated Tau (P‐Tau) and neurofibrillary tangles (NFTs) are more definitive pathologic features and are tightly linked to cognitive decline in AD patients (Caspersen et al., 2005; Reddy & Beal, 2008; Selkoe, 2001). Extracellular neuritic plaques are deposits of differently sized small peptides called β‐amyloid (Aβ) derived via sequential proteolytic cleavages of the β‐amyloid precursor protein (APP). Accumulation of Aβ has been demonstrated to occur within neurons with AD pathogenesis (Reddy et al., 2012; Ye et al., 2017; Ye & Cai, 2014).

Tau is a major microtubule‐associated protein that plays a large role in the outgrowth of neuronal processes and the development of neuronal polarity (Reddy, 2011a). Tau is abundantly present in the central nervous system and is predominantly expressed in neuronal axons. The phosphorylation of Tau regulates microtubule binding and assembly (Wang & Liu, 2008). In contrast, pathological Tau becomes hyperphosphorylated, which destabilizes microtubules by decreased binding to microtubules, resulting in the aggregation of hyperphosphorylated Tau (Brandt et al., 2005; Pradeepkiran et al., 2019; Reddy, 2011a; Reddy, 2011b). Tau can also self‐aggregate into oligomers and more extensive inclusions in neurons, known as neurofibrillary tangles (Palop & Mucke, 2009). Mitochondrial dysfunction has been strongly associated with Tau pathology in AD in recent years. Overexpression of hyperphosphorylated and aggregated Tau damages the axonal transport, leading to abnormal mitochondrial distribution (Cai & Tammineni, 2017; Cheng & Bai, 2018; Wang et al., 2009). Synapses are exposed to disease‐modified protein Tau, which may cause the loss of synaptic contacts in AD neurons (Cai & Tammineni, 2017; Du et al., 2010; Jadhav et al., 2015).

Mitochondria are intracellular organelles with key roles covering cellular metabolism and are the primary source of adenosine triphosphate (ATP) generated via oxidative phosphorylation (Camara et al., 2010; Perez Ortiz & Swerdlow, 2019). There is extensive literature supporting the role of mitochondrial dysfunction and oxidative damage in the pathogenesis of AD (Gowda et al., 2021; Han, Jeong, Sheshadri, & Cai, 2020; Han, Jeong, Sheshadri, Su, & Cai, 2020; Lin & Beal, 2006; Reddy & Beal, 2008; Swerdlow, 2018). Mitochondrial dysfunction also plays a key role in other neurodegenerative diseases such as Parkinson's disease, multiple sclerosis, Huntington's disease, and amyotrophic lateral sclerosis (ALS) (Camara et al., 2017; Reddy & Reddy, 2011).

Mitochondria are comprised of two bio‐lipid membranes: the inner membrane and the outer membrane. The inner membrane covers the mitochondrial matrix and electron transport chain, and the outer membrane is highly porous, allowing low‐molecular‐weight substances between the cytosol and the intermembrane space (Manczak & Reddy, 2012; Reddy, 2008). The outer membrane consists of a voltage‐dependent anion channel (VDAC) that provides the major pathway for transmembrane fluxes of ions and metabolites across the outer mitochondrial membrane (Colombini, 2012; Reddy, 2013).

Voltage‐dependent anion channels are abundant mitochondrial outer membrane proteins expressed in three isoforms, VDAC1‐3, and are considered “mitochondrial gatekeepers” (Sampson et al., 1996). The functions of VDACs are several‐fold, including maintaining synaptic plasticity, maintaining mitochondrial shape, regulating hexokinases (HKs) and mitochondrial interactions, and regulating apoptosis signaling (Kerner et al., 2012). VDAC1 is a crucial protein in mitochondria‐mediated apoptosis. VDAC1 forms oligomers and promotes apoptosis when the protein is overexpressed, even without any apoptotic stimulus (Reddy, 2013; Shoshan‐Barmatz et al., 2018). In addition, several recent studies revealed that VDAC proteins and their binding partners are modified post‐translationally due to VDAC hyperphosphorylation and are involved in the dysfunction of VDAC (Lemasters et al., 2012). However, the causal factors of VDAC1 phosphorylation in AD are not completely understood.

VDAC1‐phosphorylated Tau complexes block mitochondrial pores, interrupt the flux of metabolites between mitochondrial membranes and cytoplasm, and impair the gating of the VDAC channel, leading to mitochondrial dysfunction and neuronal damage in AD (Reddy, 2013). Previously, we studied tissues from human postmortem AD brains and AD mouse brains and found an abnormal interaction between phosphorylated Tau and VDAC1, suggesting a direct link between VDAC1 and phosphorylated Tau, mitochondrial dysfunction, and neuronal damage in AD (Manczak & Reddy, 2012).

Oxidative stress may activate signaling pathways that alter Tau processing and increase aberrant Tau phosphorylation by activating glycogen synthase kinase (GSK) (Lin & Beal, 2006; Verri et al., 2012). GSK3β phosphorylates VDAC1 on threonine 51, resulting in the detachment of hexokinase from VDAC1 (Pastorino et al., 2005). The binding of hexokinases with VDAC1 allows the direct access of hexokinases to mitochondrial ATP in the glycolytic pathway. Also, hexokinase inhibits apoptosis by binding to VDAC and preventing the release of cytochrome c (Abu‐Hamad et al., 2008; Azoulay‐Zohar et al., 2004).

Mitophagy is a critical mechanism in mitochondrial quality control that targets damaged mitochondria for autophagy. However, little is known about the relationship between mitophagy and pathologies in AD and other tauopathies (Jeong et al., 2022). The serine/threonine‐protein kinase (PINK1) and the E3 ubiquitin‐protein ligase (PARKIN) were recently involved in eliminating defective mitochondria by mitophagy (Cai & Jeong, 2020; Pradeepkiran et al., 2020; Sun et al., 2012). The PINK1‐PARKIN pathway plays a critical role in maintaining mitochondrial homeostasis by regulating mitophagy, mitochondrial dynamics, mitochondrial biogenesis, and mitochondria‐mediated apoptosis (Cai & Tammineni, 2016; Ham et al., 2020; Han, Jeong, Sheshadri, Su, & Cai, 2020; Ye et al., 2015). Several mitochondrial ubiquitination targets of PARKIN have been identified in mammals and flies (Sun et al., 2012). Among them are mitofusins (MFN), mitochondrial Rho GTPase 1 (Miro1), and VDAC1 (Geisler et al., 2010; Narendra et al., 2010; Wang et al., 2011; Ziviani et al., 2010). It has been speculated that ubiquitination of VDAC1 is required for mitophagy (Itakura et al., 2012). The kinase activity of PINK1 is required to target PARKIN to mitochondria subsequently (Geisler et al., 2010; Narendra et al., 2010). When PARKIN induces polyubiquitination on VDAC1, the ubiquitinated VDAC1 triggers PARKIN‐mediated mitophagy by recruiting p62/sequestosome1 (SQSTM1) and Microtubule Associated Protein 1 Light Chain 3 Beta (LC3B) to the mitochondria (Geisler et al., 2010). Several models have been proposed for how PINK1 mediates the targeting of PARKIN to mitochondria, but the mechanism is not precise.

Earlier, we extensively studied (i) VDAC1 expression in AD postmortem brains and AD mouse brain tissues (Manczak & Reddy, 2012), (ii) mitochondrial/synaptic and AD‐related genes and mitochondrial function in VDAC1^+/−^ and VDAC1^+/+^ mice (Manczak et al., 2013), (iii) RNA silencing of VDAC1 in an *in vitro* condition showing reduced levels of AD‐related genes (APP, PS1, PS2, BACE1), reduced mitochondrial fission genes (Drp1 and Fis1), increased fusion genes (Mf1, Mfn2, and Opa1), increased levels of electron transport chain genes, increased hexokinases 1 and 2 and synaptic genes (Manczak & Reddy, 2013). Based on our previous findings, in the current study, we hypothesized that a partial reduction of VDAC1 (1) reduces the interaction of phosphorylated Tau with VDAC1, (2) alters the interaction of HK1 and HK2 with VDAC1, (3) triggers PINK1‐PARKIN‐mediated mitophagy, and (4) reduces mitochondrial dysfunction and synaptic deficiencies. To support our hypothesis, in the current study, we crossed VDAC1^+/−^ mice and mutant TAU (P301L) mice and generated double mutant (VDAC1^+/−^/TAU) mice. Using cortical and hippocampal tissues from 6‐month‐old WT, VDAC1^+/−^, TAU, double mutant (VDAC1^+/−^/TAU) mice, we studied (1) hippocampal spatial learning and memory behavioral changes, (2) protein levels of mitophagy, autophagy, synaptic, and other key proteins, (3) mitochondrial structural (length and number) activity, and (4) dendritic spine count. Using VDAC1^+/−^/TAU double mutant mice, we cautiously propose that a partial reduction of VDAC1 is a potential therapeutic target for AD.

## 2 RESULTS

### 2.1 Reduced VDAC1 ameliorates TAU‐induced behavioral deficits

**FIGURE 1:**
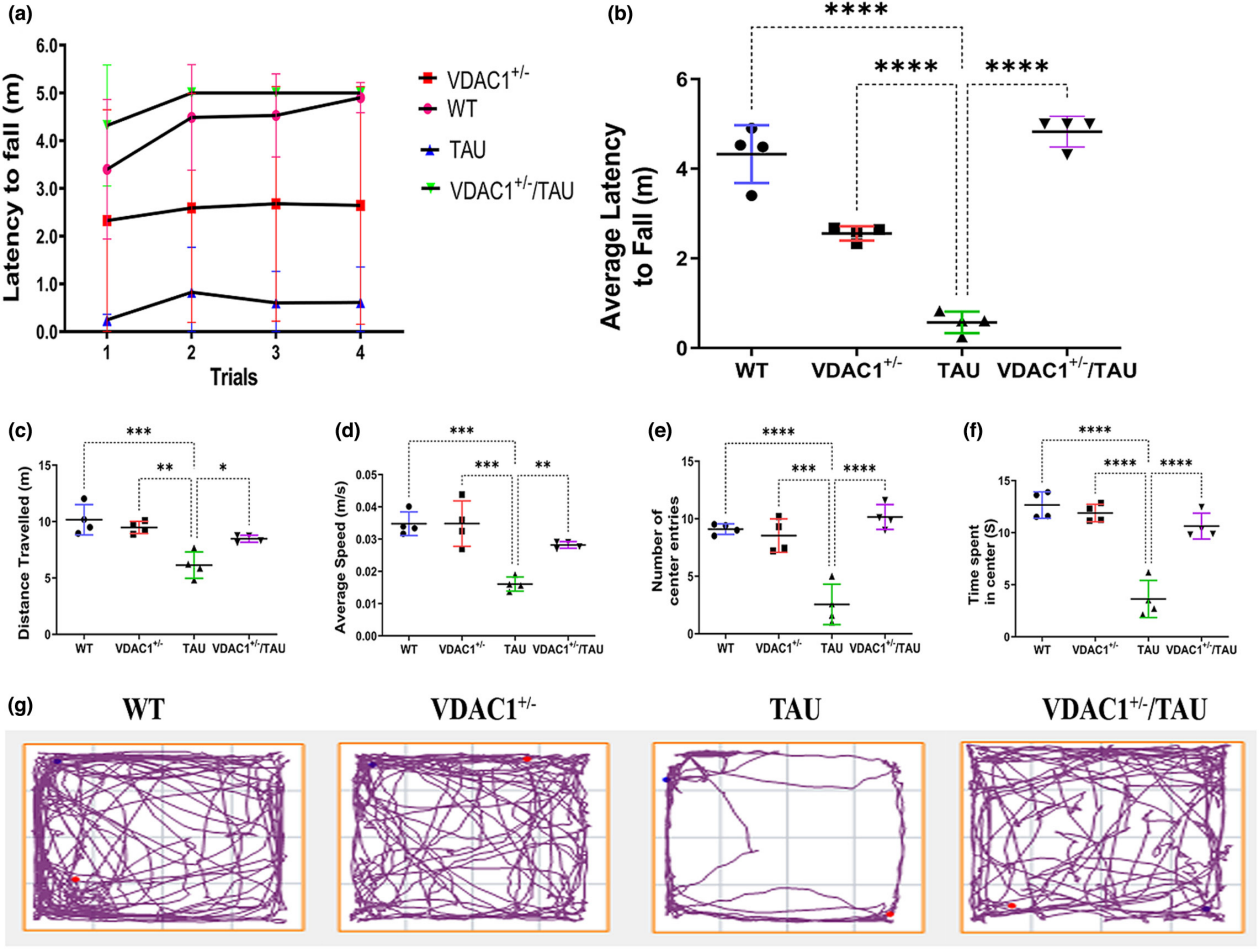
Rotarod and open field, behavioral assessment of 6‐month‐old WT, VDAC1^+/−^, TAU, and VDAC1^+/−^/TAU mice. Rotarod test: (a) latency to fall of various cohorts, namely WT, VDAC1^+/−^, TAU, and VDAC1^+/−^/TAU mice as assessed by the rotarod test. VDAC1^+/−^/TAU mice improve motor learning and coordination. (b) TAU mice spent less time on the rod (lower latency to fall), indicating impaired motor learning and coordination compared to the WT mice (****p < 0.0001). Open field test: TAU mice exhibited reduced locomotor and exploratory activity than VDAC1^+/−^/TAU mice (*p < 0.05), as evidenced by reduced total distance traveled and average speed. (c) Quantification of total distance traveled, (d) average speed, (e) the number of center entries, and (f) time spent in the center area by all indicated cohorts assessed by open field test. (g) Shows representative trajectory maps (time spent in the center) of all mentioned cohorts as analyzed by an open field test. N = 10 per group. Bars represent mean ± SEM. ns, not significant, *p <  0.05, **p  < 0.01, ***p  <  0.001, ****p < 0.0001, one‐way ANOVA followed by Turkey's test for multiple comparisons

**FIGURE 2:**
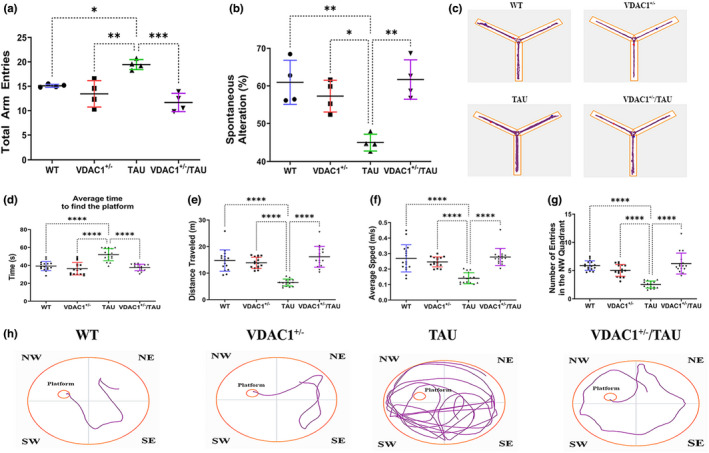
Y‐maze and Morris water maze, behavioral assessment of 6‐month‐old WT, VDAC1^+/−^, TAU, and VDAC1^+/−^/TAU mice. Y‐maze test: (a) Y‐maze test shows TAU mice display significantly more total arm entries than VDAC1^+/−^/TAU mice (***p < 0.001). (b) Spatial memory assessment using the Y‐maze spontaneous alternation test. TAU mice showed significantly reduced percentages of spontaneous alternation (**p < 0.01 vs. WT mice). VDAC1^+/−^/TAU mice significantly increased the percentage of spontaneous alternation compared with the TAU mice (**p < 0.01). (c) Representative tracks of mice (total number of entries) in the Y‐maze test. Morris water maze test: (d) the average time to find a platform was significantly decreased in VDAC1^+/−^/TAU mice compared to TAU mice (****p < 0.0001). At the same time, (e) distance traveled (****p < 0.0001), and (f) average speed (****p < 0.0001), (g) the number of entries in the NW quadrant (****p < 0.0001) were significantly increased in VDAC1^+/−^/TAU mice compared to TAU mice. (h) Representative swimming tracks of mice (time to find the platform) in Morris water maze test. *N* = 10 per group. Bars represent mean ± SEM. ns, not significant, *p < 0.05, **p < 0.01, ***p < 0.001, ****p < 0.0001, one‐way ANOVA followed by Turkey's test for multiple comparisons

Studies of the relationship between behavioral impairments and mice that overexpress human mutant TAU (P301L) suggest that mutant tau promotes the formation of phosphorylated Tau and neurofibrillary tangles, mediating age‐dependent adverse effects on memory (Lewis et al., 2000). To determine whether the reduced expression of VDAC1 ameliorates behavioral impairments in double mutant mice, using 4 widely used behavioral tests, namely the rotarod, open field, Y‐maze, and Morris Water Maze (MWM), we assessed motor coordination, locomotion, exploration abilities, spatial learning, and memory abilities. The behavior study scheme is illustrated in Figures 1 and 2. We used 6‐month‐old WT, VDAC1^+/−^, TAU, and VDAC1^+/−^/TAU mice for the above said behavioral tests.

On an accelerating rotarod test, TAU mice spent significantly less time than that WT mice (*p* < 0.0001, Figure 1a,b), validating the TAU‐induced motor deficits in mice. Relative to VDAC1^+/−^ mice, TAU mice spent less time on the rod and reached a lower maximum rate (*p* < 0.0001, Figure 1a,b), suggesting impairments in motor learning and coordination. Strikingly, the average latency to fall was increased in the VDAC1^+/−^/TAU compared with that of TAU mice (*p* < 0.0001, Figure 1a,b).

Similar to the lack of motor coordination observed in rotarod, the total distance traveled (*p* = 0.0003, Figure 1c), average speed (*p* = 0.0002, Figure 1d), number of center entries (*p* < 0.0001, Figure 1e), and time spent in center (*p* < 0.0001, Figure 1f) by TAU mice in the open field test arena was reduced significantly compared with that of WT mice affirming the TAU‐induced locomotion and exploratory impairments. Furthermore, increased locomotor and exploratory behaviors were shown by VDAC1^+/−^ mice compared with TAU mice, as evidenced by greater total distance traveled (*p* = 0.0192, Figure 1c), average speed (*p* = 0.0066, Figure 1d), number of center entries (*p* < 0.0001, Figure 1e), and time spent in center (*p* < 0.0001, Figure 1f) in a novel open field test setting. In addition, the locomotor and exploratory behavioral impairments were in VDAC1^+/−^/TAU compared with that of TAU mice as the distance traveled by mice increased significantly.

The total number of arm entries (*p* = 0.0180, Figure 2a) was increased, and the percentage of spontaneous alternation between the arms of the Y‐maze was significantly decreased (*p* = 0.0018, Figure 2b) in TAU mice as compared with WT mice suggesting impairment of spatial working memory. VDAC1^+/−^ mice had significantly less total arm entries (*p* = 0.0018, Figure 2a–c) and a higher probability of alternating three consecutive entries (*p* = 0.0121, Figure 2b) than TAU mice. Interestingly, VDAC1^+/−^/TAU mice exhibited decreased total arm entries (*p* = 0.0002, Figure 2a) and an increased percentage of spontaneous alteration (*p* = 0.0012, Figure 2b) compared with TAU mice. These results suggested that the spatial working memory of VDAC1^+/−^/TAU mice was enhanced compared with TAU mice.

TAU (P301L) mice showed an increase in time for finding the platform (*p* < 0.0001, Figure 2d), decreased distance traveled (*p* < 0.0001, Figure 2e), average speed (*p* < 0.0001, Figure 2f), number of entries in the North‐West (NW) quadrant (*p* < 0.0001, Figure 2g) compared with WT mice in the Morris Water Maze test. VDAC1^+/−^ mice showed a decrease in escape latency for finding the platform (*p* < 0.0001, Figure 2d–h), increased distance traveled (*p* < 0.0001, Figure 2e), average speed (*p* < 0.0001, Figure 2f), number of entries in the NW quadrant (*p* < 0.0001, Figure 2g) compared with TAU mice. In VDAC1^+/−^/TAU mice (*p* < 0.0001), the mean latency time for finding a platform was significantly reduced compared with TAU mice. VDAC1^+/−^/TAU mice spent more time in the NW quadrant than TAU mice (*p* < 0.0001).

Since the VDAC1^+/−^/TAU mice spent more time on the rotarod test, traveled more distance in the open field test, increased the percentage of spontaneous alteration in the Y‐maze test, and spent more time on the NW quadrant of the Morris Water Maze, we, therefore, can infer that reduced VDAC1 expression rescued the TAU‐induced motor, locomotion, and spatial memory impairments in VDAC1^+/−^/TAU mice.

### 2.2 Reduced expression of VDAC1 induces mitophagy and autophagy in VDAC1 ^+/−^/TAU mice

Recent studies on VDAC1 revealed that age and P‐Tau induced increased synaptic and mitochondrial damage, particularly abnormal regulation of mitophagy and autophagy in the disease process (Manczak & Reddy, 2012; Morton et al., 2021; Reddy & Oliver, 2019). Currently, it is unclear how reduced VDAC1 protects against defective autophagy and mitophagy.

**FIGURE 3:**
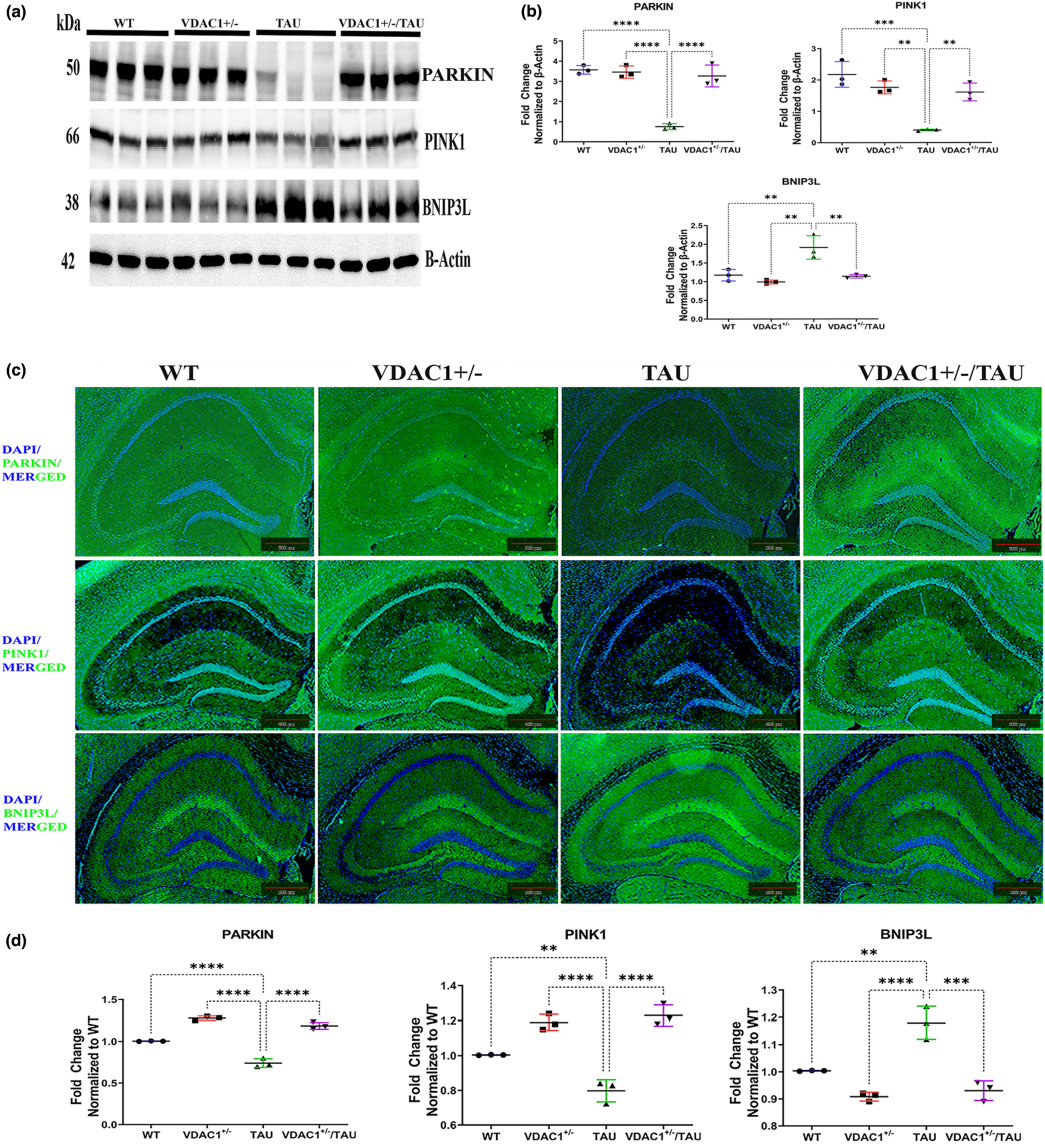
Western Blot, Immunofluorescence and quantification analysis of proteins regulating mitophagy proteins in 6‐month‐old WT, VDAC1^+/−^, TAU, and VDAC1^+/−^/TAU mice. (a) Representative immunoblots. (b) Quantitative densitometry analysis of mitophagy proteins PARKIN (****p < 0.0001), PINK1 (**p < 0.01) were significantly increased, and BNIP3L (**p < 0.01) was significantly decreased in VDAC1^+/−^/TAU mice compared to TAU mice. Each lane was loaded with 40 μg of total protein. Housing‐keeping protein beta‐actin was used as the loading control. Data are from three independent experiments with similar results (*N* = 3). (c) Representative immunofluorescence images of 10‐micron coronal sections (10×). (d) Fluorescence intensity analysis of mitophagy proteins PARKIN (****p < 0.0001), PINK1 (****p < 0.0001) were significantly increased and BNIP3L (***p < 0.001) was significantly decreased in VDAC1^+/−^/TAU mice compared to TAU mice. Data are from three independent experiments with similar results (*N* = 3) with 10–15 fields per mouse. Scale bar: 500 μm. Results were expressed as mean ± SEM. ns, not significant, *p < 0.05, **p < 0.01, ***p < 0.001, ****p < 0.0001, one‐way ANOVA followed by Turkey's test for multiple comparisons

**FIGURE 4:**
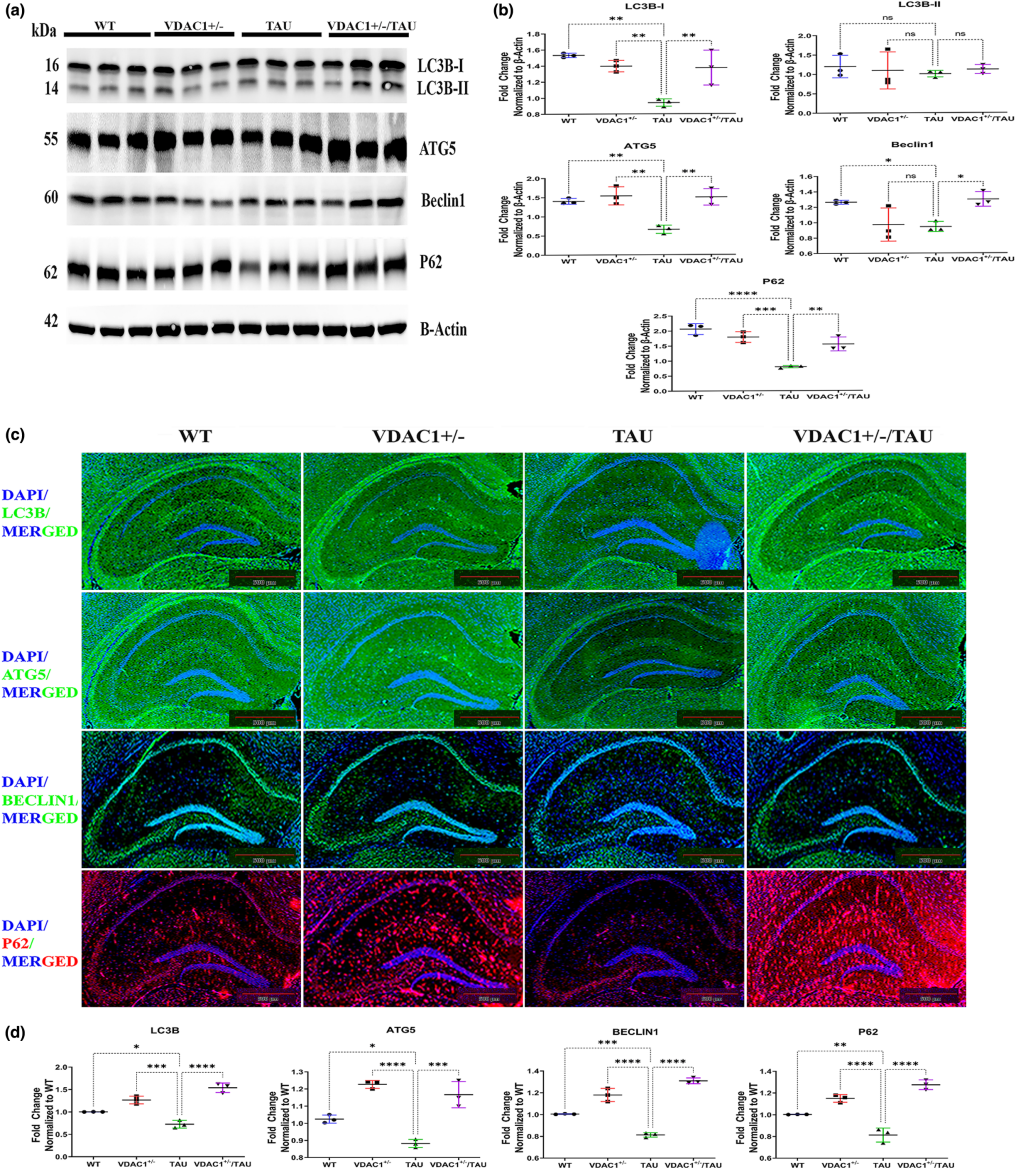
Western Blot, Immunofluorescence and quantification analysis of proteins regulating autophagy proteins in 6‐month‐old WT, VDAC1^+/−^, TAU, and VDAC1^+/−^/TAU mice. (a) Representative immunoblots. (b) Quantitative densitometry analysis of autophagy proteins‐LC3B‐I (**p < 0.01), ATG5 (**p < 0.01), Beclin1 (*p < 0.051), P62 (**p < 0.01) were significantly increased in VDAC1^+/−^ TAU mice compared to TAU mice. Each lane was loaded with 40 μg of total protein. Housing‐keeping protein beta‐actin was used as the loading control. Data are from three independent experiments with similar results (*N* = 3). (c) Representative immunofluorescence images of 10‐micron coronal sections (10×). (d) Fluorescence intensity analysis of autophagy proteins LC3B (****p < 0.0001), ATG5 (***p < 0.001), Beclin1 (****p < 0.0001), P62 (****p < 0.0001) were significantly increased in VDAC1^+/−^/TAU mice compared to TAU mice. Data are from three independent experiments with similar results (*N* = 3) with 10–15 fields per mouse. Scale bar: 500 μm. Results were expressed as mean ± SEM. ns, not significant, *p < 0.05, **p < 0.01, ***p < 0.001, ****p < 0.0001, one‐way ANOVA followed by Turkey's test for multiple comparisons

To address these issues, we crossed VDAC1 heterozygote knockout (VDAC1^+/−^) mice with transgenic TAU (P301L strain) mice and generated double mutant (VDAC1^+/−^/ TAU) mice and studied the protective effects of a partial reduction of VDAC1 on mitophagy and autophagy. We performed i) immunoblotting analysis of mitophagy and autophagy proteins from cortical tissues and ii) immunofluorescence analysis in the hippocampal sections from 6‐month‐old WT, VDAC1^+/−^, TAU, and VDAC1^+/−^/TAU mice. As a result, significantly decreased levels of mitophagy proteins (PARKIN and PINK1) and increased levels of BNIP3L were found in TAU mice (Figure 3a–d, Figure S1). In addition, decreased levels of autophagy proteins LC3BI, ATG5, Beclin1, and P62 (Figure 4a–d, Figure S2) were found in TAU mice compared with WT mice. At the same time, PARKIN, PINK1, and autophagy proteins were significantly increased in VDAC1^+/−^, VDAC1^+/−^/TAU compared with TAU mice. These results suggested that partial reduction of VDAC1 expression induces mitophagy and autophagy in VDAC1^+/−^/TAU mice.

### 2.3 Increased synaptic activity, hexokinase, AKT, and decreased phosphorylated tau, VDAC, ANT1, GSK3β levels in the VDAC1 ^+/−^/TAU mice

**FIGURE 5:**
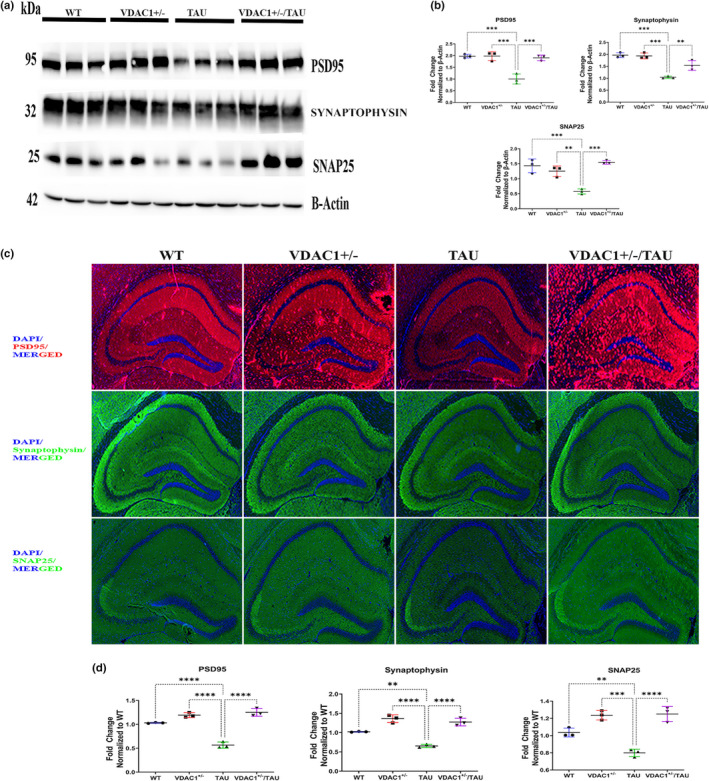
Western Blot and quantification analysis of proteins regulating synaptic proteins in 6‐month‐old WT, VDAC1^+/−^, TAU, and VDAC1^+/−^/TAU mice. (a) Representative immunoblots. (b) Quantitative densitometry analysis of synaptic proteins PSD95 (***p < 0.001), synaptophysin (**p < 0.01), SNAP25 (***p < 0.001) were significantly increased in VDAC1^+/−^/TAU mice compared to TAU mice. Each lane was loaded with 40 μg of total protein. Housing‐keeping protein beta‐actin was used as the loading control. Data are from three independent experiments with similar results (*N* = 3). (c) Representative immunofluorescence images of 10‐micron coronal sections (10×). (d) Fluorescence intensity analysis of synaptic proteins PSD95 (****p < 0.0001), synaptophysin (****p < 0.0001), SNAP25 (****p < 0.0001) were significantly increased in VDAC1^+/−^/TAU mice compared to TAU mice. Data are from three independent experiments with similar results (*N* = 3) with 10–15 fields per mouse. Scale bar: 500 μm. Results were expressed as mean ± SEM. ns, not significant, *p < 0.05, **p < 0.01, ***p < 0.001, ****p < 0.0001, one‐way ANOVA followed by Turkey's test for multiple comparisons. [Correction added on 11 July 2022, after first online publication: the layers for the beta actin panel in Figure (5a) was placed incorrectly and it has been corrected in this version.]

**FIGURE 6:**
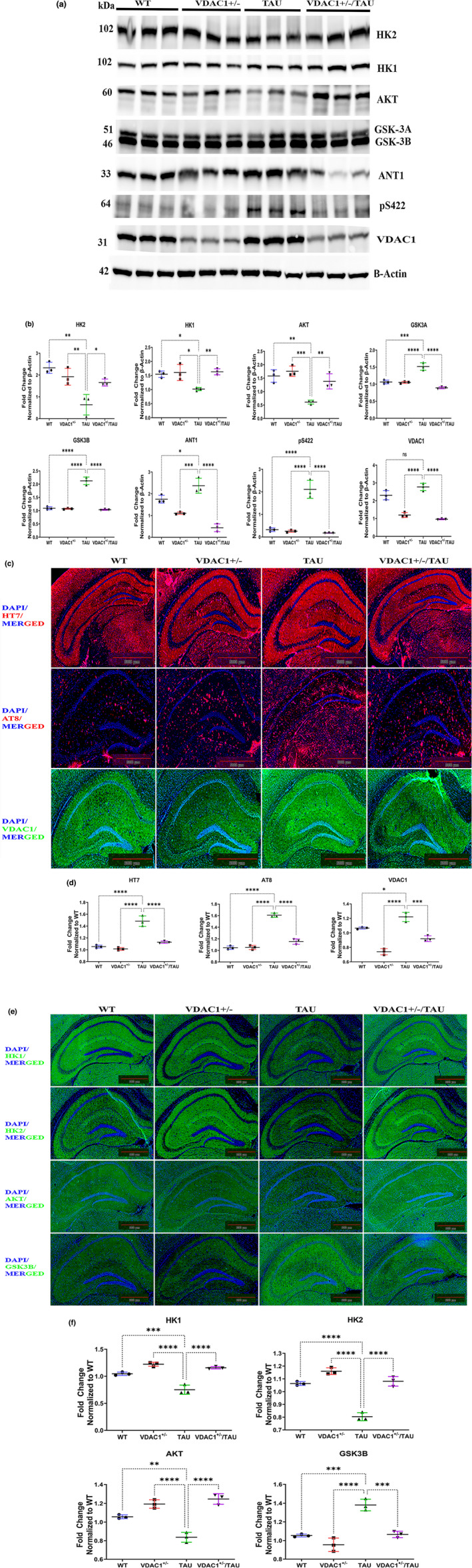
Western Blot, Immunofluorescence and quantification analysis of other key proteins (Total TAU, P‐TAU, VDAC1, HK1, HK2, AKT, GSK3B) in the hippocampal fields of 6‐month‐old WT, VDAC1^+/−^, TAU, and VDAC1^+/−^/TAU mice. (a, c, e) Representative immunoblots. (b, d, f) Quantitative densitometry analysis of HK1 (**p < 0.01), HK2 (*p < 0.05), AKT (**p < 0.01) were significantly increased, and GSK3A (****p < 0.0001), GSK3B (****p < 0.0001), ANT1 (****p < 0.0001), pS422 (****p < 0.0001), VDAC1 (****p < 0.0001) were significantly decreased in VDAC1^+/−^/TAU mice compared to TAU mice. Each lane was loaded with 40 μg of total protein. Housing‐keeping protein beta‐actin was used as the loading control. Data are from three independent experiments with similar results (*N* = 3). (c) Representative immunofluorescence images of 10‐micron coronal sections (10×). (d) Fluorescence intensity analysis of total TAU (****p < 0.0001), P‐TAU (****p < 0.0001), VDAC1 (***p < 0.001), GSK3B (***p < 0.001) were significantly decreased, HK1 (****p < 0.0001), HK2 (****p < 0.0001), AKT (****p < 0.0001) were significantly increased in VDAC1^+/−^/TAU mice compared to TAU mice. Data are from three independent experiments with similar results (*N* = 3) with 10–15 fields per mouse. Scale bar: 500 μm. Results were expressed as mean ± SEM. ns, not significant, *p < 0.05, **p < 0.01, ***p < 0.001, ****p < 0.0001, one‐way ANOVA followed by Turkey's test for multiple comparisons

The molecular pathways leading to synapse loss and dysfunction in AD are not well understood, but substantial data indicate that P‐Tau may be responsible for these effects (Jadhav et al., 2015). In the brain, hexokinase (HK) is the major isozyme present (∼70%–90%) associated with the outer mitochondrial membrane. The release of HK from mitochondria is known to cause a severe decrease in enzyme activity. Interestingly, mitochondrial‐bound hexokinase I activity in neurons has been shown to be neuroprotective, maintaining adequate glutathione levels, inducing neurite outgrowth, and preventing neuronal oxidative damage (Rose & Warms, 1967; Wang et al., 2008; Wilson, 2003). Hence, we wanted to know how a partial reduction of VDAC1 protects against hexokinases 1 and 2, detachment, and increases cellular ATP in cells. Therefore, we checked the synaptic proteins (PSD95, synaptophysin, and SNAP25), HK1, HK2, AKT, GSK3A, GSK3β, ANT, phosphorylated tau (pS422), and VDAC1 protein expression levels in the WT, VDAC1^+/−^, TAU, and VDAC1^+/−^/TAU mice. Significantly decreased levels of PSD95, synaptophysin, SNAP25, HK1, HK2, AKT, and significantly increased levels of GSK3A, GSK3β, ANT1, phosphorylated tau (pS422), VDAC1 were found in TAU mice compared with WT mice (Figures 5a–d and 6a–f, Figures S3–S4). When we analyzed the data further, PSD95, synaptophysin, SNAP25, HK1, HK2, and AKT significantly increased, and the levels of GSK3A, GSK3β, ANT1, phosphorylated tau (pS422), and VDAC1 were decreased in VDAC1^+/−^ and VDAC1^+/−^/TAU mice compared to TAU mice (Figures 5a–d, Figure 6a–f, Figures S3–S4). These observations indicate that VDAC1^+/−^ increases synaptic proteins, and hexokinase activities reduce VDAC1 and mutant and/or P‐Tau.

### 2.4 VDAC1 interaction with phosphorylated Tau, HK1, and HK2

VDAC1/ Hexokinase interactions link glycolysis and oxidative phosphorylation (Rodrigues‐Ferreira et al., 2012). HK1 binding of VDAC1 is thought to increase the catalytic efficiency of both processes by facilitating mitochondrial ATP release from VDAC for glucose phosphorylation and by channeling ADP into mitochondria for oxidative phosphorylation (Jackson et al., 2015). VDAC1 interacts with phosphorylated Tau, leading to blocking the pores of mitochondria and mitochondrial transport in AD neurons (Manczak & Reddy, 2012). In the present study, we determined whether phosphorylated Tau interacts with VDAC1, we conducted a double‐labeling analysis of VDAC1 and phosphorylated tau, VDAC1, and HK1, VDAC1, and HK2 using hippocampal sections from the brains of WT, VDAC1^+/−^, TAU, and VDAC1^+/−^/TAU mice. The interaction of VDAC1/TAU was significantly increased, and VDAC1/HK1 and VDAC1/HK2 were altered considerably in TAU mice compared with WT mice (Figures S5–S7). Further, the interaction of VDAC1 with TAU was significantly decreased, and immunoreactivity of HK1 and HK2 was altered in VDAC1^+/−^, VDAC1^+/−^/TAU mice compared with TAU mice (Figures S5–S7). In addition, VDAC1 was colocalized with phosphorylated Tau (AT8), HK1, and HK2, indicating that VDAC1 interacts with phosphorylated Tau, HK1, and HK2.

### 2.5 Mitochondrial structural alterations by TEM

**FIGURE 7:**
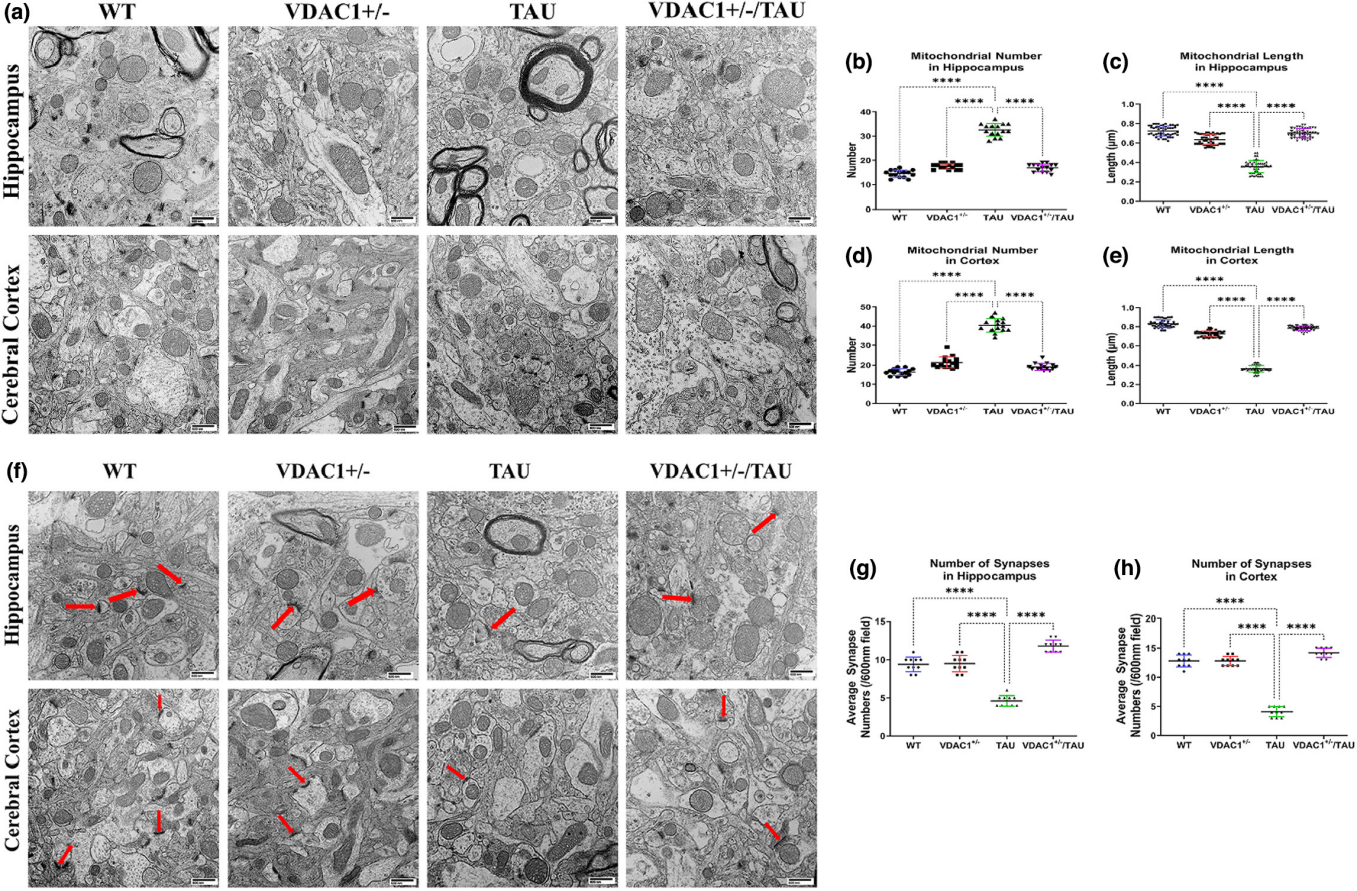
Transmission electron microscopy (TEM) analysis of the hippocampus and cerebral cortex region in 6‐month‐old WT, VDAC1^+/−^, TAU, and VDAC1^+/−^/TAU mice. (a) Representative electron micrographs of mitochondria in the hippocampus and cerebral cortex of all indicated cohorts. (b) Mitochondrial number in the hippocampus. (c) Represents the mitochondrial length in the hippocampus. (d) Represents the mitochondrial number in the cerebral cortex. (e) Represents the mitochondrial length in the cerebral cortex. Significantly decreased number of mitochondria in hippocampi (****p < 0.0001) and cortex (****p < 0.0001) of VDAC1^+/−^/TAU mice relative to TAU mice, the mitochondrial length is significantly increased in hippocampal (****p < 0.0001) and cerebral cortical tissues (****p < 0.0001) in VDAC1^+/−^/TAU mice. (f) Synaptic densities are sharply defined and contain electron‐dense materials uniformly distributed in all indicated cohorts of the hippocampus and cerebral cortex. The arrowheads show the bowl‐shaped structure of synapses and synaptic mitochondria with normal structure. Magnification ×22,000. (g) Synapse number in the hippocampus. (h) Synapse number in the cerebral cortex. The synapse numbers were found to be significantly increased in the hippocampi (****p < 0.0001) and cerebral cortical tissues (****p < 0.0001) of VDAC1^+/−^/TAU mice relative to TAU mice. *N* = 5 per group. Results were expressed as mean ± SEM. ns, not significant, *p < 0.05, **p < 0.01, ***p < 0.001, ****p < 0.0001, one‐way ANOVA followed by Turkey's test for multiple comparisons (scale bar = 600 nm)

It is well‐established that structurally damaged mitochondria are present in AD neurons and the primary neurons of AD mice, particularly at nerve terminals (Wang et al., 2014). We used transmission electron microscopy (TEM) on hippocampal and cortical tissues from 6‐month‐old WT, VDAC1^+/−^, TAU, and VDAC1^+/−^/TAU mice to determine the effects of VDAC1^+/−^ on mitochondrial number and length. As shown in Figure 7a, we observed a substantial increase in the number of mitochondria in 6‐month‐old TAU mice hippocampi (*p* < 0.0001) compared with WT mice (Figure 7b). We also assessed mitochondrial length in TAU mice; the mitochondrial length was significantly decreased (*p* < 0.0001) compared with WT mice (Figure 7c). On the contrary, the mitochondrial number was significantly decreased, and mitochondrial length was increased in VDAC1^+/−^ (Figure 7b) and VDAC1^+/−^/TAU (Figure 7c) mice hippocampi compared with TAU (P301L) mice. As illustrated in Figure 7a, there was a substantial increase in the number of mitochondria in 6‐month‐old TAU mice cortical tissues (*p* < 0.0001) compared with WT mice (Figure 7d). We also assessed mitochondrial length, we observed that in TAU mice, mitochondrial length was significantly decreased (*p* < 0.0001) compared with WT mice (Figure 7e). On the contrary, the mitochondrial number was significantly decreased, and mitochondrial length was increased in VDAC1^+/−^ (Figure 7d) and VDAC1^+/−^/TAU (Figure 7e) mice cortical tissues compared with TAU mice.

### 2.6 Impact of VDAC1 ^+/−^ on synapse numbers in the hippocampal and cortical tissues

It is well‐established that spine density is critical for synaptic function and cognitive behavior in AD patients and AD mice (Manczak et al., 2018). Therefore, we also examined the impact of VDAC1^+/−^ on synapse organization and numbers in both hippocampal and cortical tissues of WT, VDAC1^+/−^, TAU, and VDAC1^+/−^/TAU mice. The synapse location and organization in the red arrow showed the synaptic cleft. As shown in Figure 7f, the average synapse numbers were significantly decreased in TAU mice hippocampi (Figure 7g) and cortex (Figure 7h) relative to WT mice. At the same time, the average synapse numbers were significantly increased in VDAC1^+/−^, VDAC1^+/−^/TAU mice hippocampi, and cortex relative to TAU mice (Figure 7g,h).

### 2.7 Reduced expression of VDAC1 increases the dendritic spine density

**FIGURE 8:**
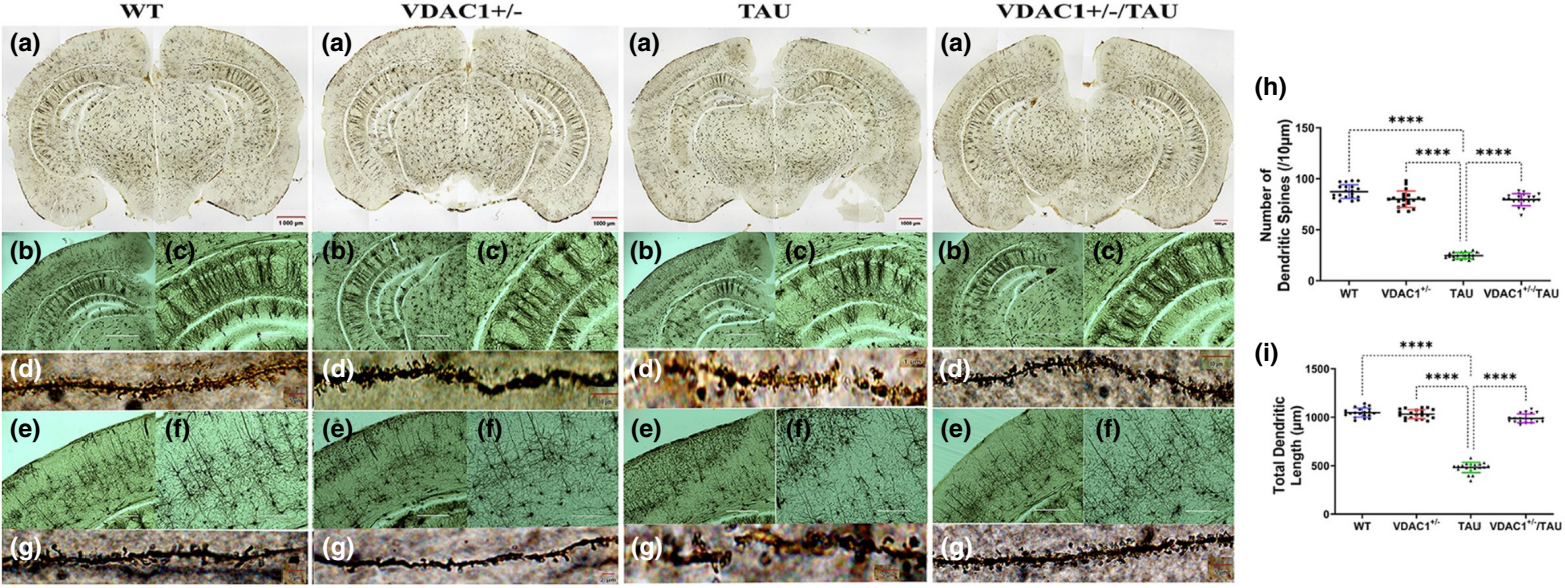
Microphotography of Golgi‐Cox impregnated brain slice of 6‐month‐old WT, VDAC1^+/−^, TAU, and VDAC1^+/−^/TAU mice. (a) Golgi‐Cox impregnated image of the whole mouse brain. (b, c) Images of the hippocampus (4× and 10×) are well stained. (d) Magnified images of the hippocampus‐ Dendritic spines can also be visualized at higher magnification (100×). (e, f) The cerebral cortex (10× and 20×) is well stained. (g) Magnified images of the cerebral cortex‐ Dendritic spines can also be visualized at higher magnification (100×). (h) The number of dendritic spines in the hippocampus. (i) Dendritic length in the hippocampus. Significantly increased dendritic number (****p < 0.0001) and the length of dendritic spines (****p < 0.0001) in VDAC1^+/−^/TAU mice relative to TAU mice. *N* = 5 per group. Results were expressed as mean ± SEM. ns, not significant, *p < 0.05, **p < 0.01, ***p < 0.001, ****p < 0.0001, one‐way ANOVA followed by Turkey's test for multiple comparisons. Scale bars 1000 μm in (a, b & e), 400 μm in (c), 200 μm in (f) and 1 μm, 2 μm, 5 μm, 10 μm in (d & g)

We quantified dendritic length and the number of spines using Golgi‐cox staining in the hippocampus and cortex of 6‐month‐old WT, VDAC1^+/−^, TAU, and VDAC1^+/−^/TAU mice to assess the effects of VDAC1^+/−^ on dendritic length and spines. Figure 8a–g showed the representative images of dendrites in four different groups of mouse brains covering both cortex and hippocampus areas at 4× (Figure 8a), 10× (Figure 8b–e), 20× (Figure 8c–f), and 100× (Figure 8d–g) magnifications. Hippocampal and cortical neurons from TAU mice showed a significant visual difference with reduced length and number of dendrites compared with WT mice (Figure 8h–i). In addition, the measurement of dendritic length and the number of dendritic spines showed a significant difference in VDAC1^+/−^ and VDAC1^+/−^/TAU mice relative to TAU mice in both hippocampal and cortical neurons (Figure 8h–i). These results confirmed the significant positive impact of VDAC1^+/−^ on hippocampal and cortical neurons, dendritic morphology, and quality.

## 3 DISCUSSION

Mitochondrial dysfunction is a common pathological feature and contributes to neurodegeneration in AD (Reddy & Beal, 2008). Recent reports suggested that mitochondrial fission/fusion, biogenesis, mitophagy, and autophagy are altered in postmortem AD brains and both *in vitro* and *in vivo* models of disease (Calkins et al., 2011; Fang et al., 2019; Kandimalla et al., 2016; Kandimalla et al., 2018; Kerr et al., 2017; Manczak et al., 2011; Reddy, 2014; Reddy et al., 2018; Santos et al., 2010; Su et al., 2010; Swerdlow et al., 2014; Wang et al., 2009). Other researchers and we previously reported that Aβ and P‐Tau interact with many mitochondrial proteins (DRP1 and VDAC1), leading to mitochondrial dysfunction and the depletion of major mitophagy and autophagy proteins (Hirai et al., 2001; Ishihara et al., 2009; Kshirsagar et al., 2021; Manczak et al., 2010; Reddy et al., 2012; Reddy et al., 2018; Shoshan‐Barmatz et al., 2018). In addition, our laboratory previously reported that reduced levels of VDAC1 may lead to decreased interaction between VDAC1 and APP, Aβ, and phosphorylated Tau and may allow mitochondrial pore opening and pore closure, ultimately leading to normal mitochondrial function and synaptic ATP and boosting synaptic and cognitive functions in AD (Manczak & Reddy, 2012). We also found that VDAC1^+/−^ mice showed improved mitochondrial function and synaptic activity and reduced expressions of several AD‐related genes compared with VDAC1^+/+^ mice (Manczak et al., 2013). Further, we reported that reducing the human VDAC1 gene in an *in vitro* condition might enhance synaptic activity, improve mitochondrial maintenance and function, and protect against AD‐related genes' toxicities (Manczak & Reddy, 2013). In the current study, we have provided *in vivo* evidence that partial reduction of VDAC1 mediated mitophagy, autophagy, synaptic, reduced P‐Tau pathology, and lessened memory impairment and anxiety symptoms in a murine model.

Our overall analysis of 6‐month‐old WT, VDAC1^+/−^, TAU, and VDAC1^+/−^/TAU mice revealed that reduced protein levels of all mitophagy, autophagy, synaptic, and other key proteins (HK1, HK2, AKT) and increased other key proteins (GSK3A, GSK3β, ANT1, pS422, VDAC1) in TAU mice compared to WT mice. In contrast, we observed the reverse trend in VDAC1^+/−^ and VDAC1^+/−^/TAU mice compared with TAU (P301L) mice. Current findings of increased protein levels of mitochondrial and synaptic genes agree with our earlier observations (Manczak & Reddy, 2012).

Cognitive impairment and anxiety are extensively reported in most AD patients (Goncalves et al., 2020; Teri et al., 1999) and are associated with increased conversion rates from MCI to AD (Mah et al., 2015). Here, we show that 6‐month‐old TAU (P301L) mice displayed impaired behavior in the rotarod, Y‐maze, open field, and Morris Water Maze behavioral tests. In addition, TAU mice have deficient motor learning and coordination activities compared with WT mice. Briefly, TAU mice could not stand on the rotarod for a longer time. At the same time, VDAC1^+/−^and VDAC1^+/−^/TAU mice stayed longer on the rotarod than TAU mice.

Furthermore, TAU mice exhibited decreased locomotor and exploratory behavior, as evidenced by decreased total distance traveled, average speed, number of center entries, and time spent in the center in a novel open field test setting. On the contrary, VDAC1^+/−^and VDAC1^+/−^/TAU mice are substantially more involved in exploring the field than TAU mice. Similar to learning and motor coordination activities, VDAC1^+/−^and VDAC1^+/−^/TAU mice did well on spatial recognition and working memory, as evidenced by the Y‐maze test. In addition, the percentage of spontaneous alteration was significantly higher in the VDAC1^+/−^and VDAC1^+/−^/TAU mice than in TAU mice. Most importantly, hippocampal‐dependent learning and memory were increased dramatically in VDAC1^+/−^and VDAC1^+/−^/TAU mice relative to TAU mice. This data strongly suggests that reduced expression of VDAC1 has improved hippocampal‐dependent learning and memory.

Mitophagy is the selective elimination of damaged mitochondria and is thus essential for mitochondrial quality control (Ashrafi & Schwarz, 2013). A recent paper has shown that VDAC1 is indeed one of the regulators of mitophagy (Ordureau et al., 2018). But others are still debating whether VDAC1 is a critical component for the PINK1‐PARKIN pathway or VDAC1 is irrelevant to mitophagy (Ham et al., 2020). Here, we identify PINK1, PARKIN, p62, and the mitochondrial substrate VDAC1 as key players in a sequential mitophagy process in VDAC1^+/−^/TAU double mutant mice. These observations support that the partial reduction of VDAC1 activates mitophagy and reduces excessive mitochondrial fragmentation in VDAC1^+/−^/TAU mice. Furthermore, we observed a mechanistic link between reduced VDAC1‐dependent enhanced mitophagy and autophagy in AD for the first time.

Hexokinase is the key enzyme in glucose metabolism. The decreased glucose metabolism in TAU (P301L) mice reflects the abnormal expression and distribution of HK (Chiara et al., 2008; Pastorino & Hoek, 2008). HK1 and HK2 are mitochondrial hexokinase isotypes because they participate in glucose metabolism by binding to mitochondria. HK1 and HK2 protein levels were reduced in 3XTg AD mice (Han et al., 2021). Hexokinase isoforms bind to mitochondrial outer membranes in large part by interacting with the outer membrane VDAC1 (Pastorino & Hoek, 2008). GSK3β phosphorylates VDAC1 on threonine 51, resulting in the detachment of hexokinase from VDAC1. Given the association of GSK3 with phosphorylation in AD, VDAC1 is phosphorylated on the putative GSK3β epitope in AD, leading to the inability of hexokinases to interact with VDAC1, resulting in the dissociation of VDAC1 from hexokinases (Pastorino et al., 2005).

Abnormalities in mitochondrial pore opening and closure may lead to defects in oxidative phosphorylation, mitochondrial dysfunction, and ultimately cell death (Manczak et al., 2013). Several key mitochondrial proteins, including outer membrane protein VDAC1, inner membrane protein ANT, and matrix protein Cyclophilin D (CypD), are involved in mitochondrial pore opening and pore closure (Manczak et al., 2013). We previously reported that Hexokinases 1 and 2 were significantly upregulated in the VDAC^+/−^ mice (Manczak & Reddy, 2013). Further, free radical production and lipid peroxidation levels were reduced in the VDAC1^+/−^ mice, and cytochrome oxidase activity and ATP levels were elevated, indicating an enhanced mitochondrial function in the VDAC1^+/−^ mice (Manczak & Reddy, 2013). Our present study found increased protein expression of HK1, HK2, and AKT and decreased GSK3A, GSK3β, and ANT1 in VDAC1^+/−^ heterozygote knockout and VDAC1^+/−^/TAU double mutant mice. We also found reduced phosphorylated Tau (pS422) protein levels in 6‐month‐old VDAC1^+/−^ and age‐matched double mutant VDAC1^+/−^/TAU mice relative to TAU mice. Our study understands how partial reduction of VDAC1 impacts HK1 and HK2 and glycolic pathways in TAU mice in disease progression. Further studies are still needed to understand the mechanistic links.

We previously studied the RNA silencing of VDAC1 and assessed mitochondrial function in AD pathogenesis (Manczak & Reddy, 2013). We reported increased mRNA expression of synaptic function and mitochondrial fission genes and reduced levels of mitochondrial fusion genes in RNA‐silenced SHSY5Y cells for VDAC1 gene. In addition, RNA‐silenced VDAC1 gene in SHSY5Y cells showed reduced H_2_O_2_ production, lipid peroxidation, and fission‐linked guanosine triphosphate (GTPase) activity, and increased cytochrome oxidase activity and ATP production (Manczak & Reddy, 2013). In the present study, increased expression of synaptic genes suggests that reduced VDAC1 is beneficial in the presence of Tau in double mutant (VDAC1^+/−^/TAU) mice. These observations demonstrated that phosphorylated Tau interaction with VDAC1 increases mitochondrial fragmentation, ultimately leading to mitochondrial dysfunction and neuronal damage. Findings from our current study support our previous study, in which increased VDAC1 levels correlated with reduced synaptic and mitochondrial activity at different stages in disease progression (Manczak & Reddy, 2012). Taken together, these findings suggest that partial reduction of VDAC1 may be beneficial to the maintenance of mitophagy, autophagy, and synaptic activity.

Dysfunction of mitochondria is correlated with disease progression in neurodegenerative diseases and is suggested to contribute to excessive neuron loss in AD (Chan, 2006; Cipolat et al., 2006; Wu et al., 2014). We found significant differences in the mitochondrial length and number in the hippocampal and cortical tissues of 6‐month‐old VDAC1^+/−^, TAU, and VDAC1^+/−^/TAU mice relative to age‐matched WT mice. There were increased numbers of large, abnormal‐shaped mitochondria in the TAU mice. Furthermore, we also found disrupted cristae in the TAU mice. In addition, mitochondrial length was drastically decreased in the TAU mice compared with WT mice in both the hippocampus and cortex. Oxygen tension, oxidative stress, and autophagic activation are the factors that can modulate the mitochondrial shape (Gomes et al., 2011). On the contrary, increased mitochondrial number and decreased length in TAU (P301L) mice may be due to excessive mitochondrial fragmentation or ineffective degradation of damaged mitochondria after fission. A similar observation was reported in an earlier study of P301L mice (Kandimalla et al., 2016; Kandimalla et al., 2018). In VDAC1^+/−^ and VDAC1^+/−^/TAU mice, we observed a decreased mitochondrial number, in other words, reduced expression of VDAC1 suppresses mitochondrial fragmentation. The mitochondrial length was significantly increased in both hippocampus and cortical tissues of VDAC1^+/−^ and VDAC1^+/−^/TAU mice relative to TAU mice. These observations indicate that reduced expression of VDAC1 balances mitochondrial dynamics. AD has been proposed to result from synaptic connections and plasticity defects in the hippocampus and cortex (Manczak et al., 2018; Reddy et al., 2018). The morphological analysis revealed altered synaptic density and morphology in the TAU mice in both hippocampus and cortex. The decrement in synapse numbers may be due to the toxic effects of phosphorylated Tau. Whereas in VDAC1^+/−^ and VDAC1^+/−^/TAU mice, synapse numbers were drastically increased compared with TAU (P301L) mice. Based on these observations, we cautiously conclude that the quality of mitochondria, synapses are improved in VDAC1^+/−^/TAU mice.

Dendritic spines bear a strong potential for morphological plasticity, thus enabling neurons to modify their synaptic interconnections, the correlates of learning and memory (Hoffmann et al., 2013). Quantitative dendritic spine analysis is crucial in AD research as there is principal evidence that the disease changes the number, structure, and function of these postsynaptic sites of excitatory synapses (John & Reddy, 2021; Kandimalla et al., 2018; Kartalou et al., 2020; Reddy & Beal, 2008). Our earlier findings stated that a reduced number of dendritic spines and synaptic proteins are extensively reported in mouse models of AD (Hegde et al., 2019; Kandimalla et al., 2016) and postmortem AD brains (Reddy et al., 2005; Reddy & Beal, 2008). These observations prompted an investigation of VDAC1^+/−^'s impact on dendritic spines in TAU (P301L) mice. As described above, we quantified dendritic length and a number of spines using Golgi‐Cox staining in the hippocampus and cortex of 6‐month WT, VDAC1^+/−^, TAU, and VDAC1^+/−^/TAU mice. We observed significantly increased dendritic length and number in VDAC1^+/−^and VDAC1^+/−^/TAU mice relative to TAU mice. Our study observations strongly indicate that the reduced expression of VDAC1 enhances both dendritic length and the number of dendritic spines in VDAC1^+/−^/TAU mice. Our dendritic number and length observations positively correlated with improved behavior in VDAC1^+/−^ and VDAC1^+/−^/TAU mice.

In conclusion, we have demonstrated that the partial reduction of VDAC1 improves (i) spatial learning and memory, (ii) mitophagy, autophagy, synaptic, and other key proteins, (iii) mitochondrial length and number, and (iv) synapse and dendritic spine morphology in VDAC1^+/−^, and VDAC1^+/−^/TAU mice. Our findings further suggest that the impaired mitophagy in TAU contributes to an accumulation of defective mitochondria with abnormal morphology, causing mitochondrial dysfunction and a deficiency in cellular energy supply. Further, our current study observations provided protective effects of reduced VDAC1 against P‐Tau‐induced mitochondrial and synaptic toxicities in TAU (P301L) mice and provided new evidence to develop VDAC1 therapeutic strategies for AD. Ours is the first genetic crossing study to report the beneficial effects of reduced VDAC1 in AD.

## 4 METHODS

### 4.1 Animals

To study the partial reduction of VDAC1, we used VDAC1^+/−^ heterozygote knockout mice and mutant TAU mice (P301L line). TAU mice were generated with human Tau P301L mutation (Lewis et al., 2000), and VDAC1^+/−^ mice generation has been described previously (Weeber et al., 2002). Homozygous VDAC1^−/−^ knockout mice are partially embryonic lethal depending on the strain background (Weeber et al., 2002). However, heterozygote VDAC1^+/−^ mice are viable, fertile, normal size, and do not show any phenotypic abnormalities (Manczak et al., 2013). TAU mice were purchased from Taconic Biosciences. We generated the double mutant (VDAC1^+/−^/TAU) mice by genetic crossing VDAC1^+/−^ mice with TAU mice. We genotyped the VDAC1^+/−^ and TAU mutations using DNA prepared from tail biopsy and PCR amplification, as described earlier (Lewis et al., 2000; Weeber et al., 2002). We used both male and female mice for this study. Mice were bred and housed under a standard 12 h light–dark cycle, with lights on at 7 AM in the Laboratory Animal Resource Center, Texas Tech University Health Sciences Center, accredited by the Association for Assessment and Accreditation of Laboratory Animal Care International (AAALAC). All experimental protocols were approved by Texas Tech University Health Sciences Center—Institutional animal care and use committee (TTUHSC‐IACUC).

### 4.2 Behavior tests, immunoblotting, and immunofluorescence analysis

We performed behavioral studies to examine the motor balance, coordination, motor planning, locomotor activity levels, spatial learning, and memory, along with immunoblotting and immunofluorescence analysis as previously described (Hegde et al., 2019; Vijayan, Bose, & Reddy, 2021a; Vijayan, Bose, & Reddy, 2021b; Vijayan, George, et al., 2021) (Appendix S1).

### 4.3 TEM of brain mitochondria

To determine the mitochondrial number and size, we performed transmission electron microscopy in hippocampal and cortical sections of 6‐month‐old WT, VDAC1^+/−^, TAU, and VDAC1^+/−^/TAU mice. Animals were perfused using the standard method, and the brains were removed from the mice as described earlier (Vijayan, Bose, & Reddy, 2021a; Vijayan, Bose, & Reddy, 2021b). Briefly, the ventral part of the hippocampus layer‐the CA1 region and cerebral cortex were isolated and cut into ~1 mm^3^ cubes. Tissues were fixed in a solution of 0.1 M cacodylate buffer, 1.5% paraformaldehyde, and 2.5% glutaraldehyde and then post‐fixed with 1% osmium tetroxide and embedded in LX‐112 resin. Ultrathin sections were cut, stained with uranyl acetate and lead citrate, and examined with a Hitachi H‐7650 /Transmission Electron Microscope at 60 kV located at the College of Arts and Sciences Microscopy, Texas Tech University. Low‐magnification imaging was followed by high‐magnification imaging. Representative images were acquired and recorded with an AMT digital camera. Analyses of mitochondria number and size in WT, VDAC1^+/−^, TAU, and VDAC1^+/−^/TAU mouse brains were performed using Image J software. Briefly, mitochondria within a defined area of the field were identified and numbered by two independent, experienced researchers blinded from the details of each sample group. For mitochondria number and size measurement, 15 random micrographs were taken from the hippocampus and cerebral cortex of WT, VDAC1^+/−^, TAU, and VDAC1^+/−^/TAU mice (Vijayan, Bose, & Reddy, 2021a; Vijayan, Bose, & Reddy, 2021b).

### 4.4 Dendrite and spine analysis in Golgi‐Cox‐stained slices

Dendritic spines of neurons in the brains of 6‐month‐old WT, VDAC1^+/−^, TAU, and VDAC1^+/−^/TAU mice were detected by Golgi‐Cox staining, which was performed using the FD Rapid GolgiStain Kit (PK401, FD NeuroTechnologies) as described earlier (Hegde et al., 2019; Vijayan, Bose, & Reddy, 2021a; Vijayan, Bose, & Reddy, 2021b). All procedures were performed under dark conditions. Mouse brain tissues were impregnated for 2 weeks and processed according to the manufacturer's instructions as in our recent publications (Hegde et al., 2019; Vijayan, Bose, & Reddy, 2021a; Vijayan, Bose, & Reddy, 2021b). Briefly, dendrites within the CA1 subregion of the hippocampus and cerebral cortex were imaged using a 4×, 10×, 20×, and 100× objective using EVOS microscope‐AMG (thermofisher.com) and Olympus1X83. Approximately 20 neurons were randomly selected from each group and quantified with a double‐blind, controlled design. In addition, ImageJ and Image‐Pro Plus were used to evaluate the number of spines and the total dendritic length.

### 4.5 Statistical analysis

Data were represented as mean ± standard error of the mean (SEM). Conclusions were drawn based on statistical analyses using GraphPad™ PRISM software (version 9.0; GraphPad Software). The one‐way ANOVA was performed using Tukey's test for multiple comparisons. Group comparisons were considered significant when the *p*‐value was less than 0.05 (*p* < 0.05).

For all other procedures, see Appendix S1.

## AUTHORS CONTRIBUTIONS

P.H.R. and M.V contributed to the conceptualization and formatting of the article. M.V performed Western blot, immunofluorescence, TEM, and Golgi‐Cox experiments. R.V.A., RZ.V.A., and L.E.B performed immunofluorescence staining. J.P.A performed behavioral analysis. M.V analyzed Western blot, immunofluorescence, TEM, Golgi‐Cox, and behavioral data. M.V. and P.H.R are responsible for writing, original draft preparation, and finalizing the manuscript. P.H.R is responsible for funding acquisition.

## CONFLICT OF INTEREST

The authors declare no conflict of interest.

## Supporting information


Table S1–S2

Figure S1–S7

Appendix S1

## Data Availability

The data that support the findings of this study are available from the corresponding author upon reasonable request.
