# Supplementary material for: A partial reduction of VDAC1 enhances mitophagy, autophagy, synaptic activities in a transgenic Tau mouse model
Source: Aging Cell. 2022 Jul 7;21(8):e13663. doi: 10.1111/acel.13663 (PMC9381918; doi:10.1111/acel.13663)
Supplement: Supplementary file 1 — Table S1–S2 [file ACEL-21-e13663-s001.docx]

**Supplemental Table 1. Summary of antibody dilutions used in the immunoblotting analysis of mitophagy, autophagy, synaptic, and other key proteins in 6-months-old WT, VDAC1^+/-^, TAU, and VDAC1^+/-^/TAU mice**

| **Protein** | **Catalog Numbers** | **Primary antibody-species and dilution** | **Purchased from the company,**  **city and state** | **Secondary antibody, dilution** |
| --- | --- | --- | --- | --- |
| PARKIN | 4211 | Rabbit Polyclonal 1:1000 | Cell Signaling Technology | Donkey anti-rabbit HRP 1:10000 |
| PINK1 | ab23707 | Rabbit Polyclonal 1:500 | Abcam | Donkey anti-rabbit HRP 1:10000 |
| BNIP3L | 12396 | Rabbit Polyclonal 1:1000 | Cell Signaling Technology | Donkey anti-rabbit HRP 1:10000 |
| LC3B | 83506 | Rabbit Polyclonal 1:1000 | Cell Signaling Technology | Donkey anti-rabbit HRP 1:10000 |
| ATG5 | 12994 | Rabbit Polyclonal 1:1000 | Cell Signaling Technology | Donkey anti-rabbit HRP 1:10000 |
| Beclin1 | 3495 | Rabbit Polyclonal 1:1000 | Cell Signaling Technology | Donkey anti-rabbit HRP 1:10000 |
| P62 | H00008878-M01 | Mouse Monoclonal 1:400 | Abnova | Sheep anti-mouse HRP 1:10000 |
| PSD95 | 3450 | Rabbit Polyclonal 1:1000 | Cell Signaling Technology | Donkey anti-rabbit HRP 1:10000 |
| SYNAPTOPHYSIN | NBP2-25170 | Rabbit Polyclonal 1:3000 | Novus Biologicals | Donkey anti-rabbit HRP 1:10000 |
| SNAP25 | NBP1-88769 | Rabbit Polyclonal 1:1000 | Novus Biologicals | Donkey anti-rabbit HRP 1:10000 |
| HK1 | 2024 | Rabbit Polyclonal 1:1000 | Cell Signaling Technology | Donkey anti-rabbit HRP 1:10000 |
| HK2 | 22029-1-AP | Rabbit Polyclonal 1:2000 | Protein Tech Group | Donkey anti-rabbit HRP 1:10000 |
| AKT | 9272 | Rabbit Polyclonal 1:1000 | Cell Signaling Technology | Donkey anti-rabbit HRP 1:10000 |
| GSK3A/B | 5676 | Rabbit Polyclonal 1:1000 | Cell Signaling Technology | Donkey anti-rabbit HRP 1:10000 |
| ANT1 | ab110322 | Rabbit Polyclonal 1:1000 | Abcam | Donkey anti-rabbit HRP 1:10000 |
| P-TAU | 4BDX-1501 | Mouse Monoclonal 1:1000 | 4BioDx | Sheep anti-mouse HRP 1:10000 |
| VDAC1 | NB100-695 | Rabbit Polyclonal 1:500 | Novus Biologicals | Donkey anti-rabbit HRP 1:10000 |
| B-Actin | A2228 | Mouse Monoclonal 1:2000 | Millipore Sigma | Sheep anti-mouse HRP 1:10000 |

**Supplemental Table 2. Summary of antibody dilutions used in the immunofluorescence analysis of mitophagy, autophagy, synaptic, and other key proteins in 6-months-old WT, VDAC1^+/-^, TAU, and VDAC1^+/-^/TAU mice**

| **Protein** | **Catalog Numbers** | **Primary antibody-species and dilution** | **Purchased from the company,**  **city and state** | **Secondary antibody, dilution** |
| --- | --- | --- | --- | --- |
| PARKIN | NBP2-67017 | Rabbit Polyclonal 1:100 | Novus Biologicals | Goat anti-rabbit Alexa Flour 488 1:200 |
| PINK1 | ab23707 | Rabbit Polyclonal 1:100 | Abcam | Goat anti-rabbit Alexa Flour 488 1:200 |
| BNIP3L | 12396 | Rabbit Polyclonal 1:100 | Cell Signaling Technology | Goat anti-rabbit Alexa Flour 488 1:200 |
| LC3B | 83506 | Rabbit Polyclonal 1:50 | Cell Signaling Technology | Goat anti-rabbit Alexa Flour 488 1:200 |
| ATG5 | NBP2-54702 | Rabbit Polyclonal 1:100 | Novus Biologicals | Goat anti-rabbit Alexa Flour 488 1:200 |
| Beclin1 | NB500-249 | Rabbit Polyclonal 1:100 | Novus Biologicals | Goat anti-rabbit Alexa Flour 488 1:200 |
| P62 | H00008878-M01 | Mouse Monoclonal 1:50 | Abnova | Goat anti-mouse Alexa Flour 594 1:200 |
| PSD95 | MA1-046 | Mouse Monoclonal 1:250 | Invitrogen | Goat anti-mouse Alexa Flour 594 1:200 |
| SYNAPTOPHYSIN | NBP2-25170 | Rabbit Polyclonal 1:250 | Novus Biologicals | Goat anti-rabbit Alexa Flour 488 1:200 |
| SNAP25 | NBP1-88769 | Rabbit Polyclonal 1:100 | Novus Biologicals | Goat anti-rabbit Alexa Flour 488 1:200 |
| HK1 | 2024 | Rabbit Polyclonal 1:100 | Cell Signaling Technology | Goat anti-rabbit Alexa Flour 488 1:200 |
| HK2 | 22029-1-AP | Rabbit Polyclonal 1:100 | Protein Tech Group | Goat anti-rabbit Alexa Flour 488 1:200 |
| AKT | 9272 | Rabbit Polyclonal 1:100 | Cell Signaling Technology | Goat anti-rabbit Alexa Flour 488 1:200 |
| GSK3A/B | 5676 | Rabbit Polyclonal 1:100 | Cell Signaling Technology | Goat anti-rabbit Alexa Flour 488 1:200 |
| P-TAU (AT8) | MN1020 | Mouse Monoclonal 1:100 | Thermo Scientific | Goat anti-mouse Alexa Flour 594 1:200 |
| VDAC1 | 55259-1-AP | Rabbit Polyclonal 1:100 | Protein Tech Group | Goat anti-rabbit Alexa Flour 488 1:200 |
| VDAC1 | ab14734 | Mouse Monoclonal 1:100 | Abcam | Goat anti-mouse Alexa Flour 594 1:200 |
