# Supplementary material for: A partial reduction of VDAC1 enhances mitophagy, autophagy, synaptic activities in a transgenic Tau mouse model
Source: Aging Cell. 2022 Jul 7;21(8):e13663. doi: 10.1111/acel.13663 (PMC9381918; doi:10.1111/acel.13663)
Supplement: Supplementary file 2 — Figure S1–S7 [file ACEL-21-e13663-s003.docx]

**Supplementary Figure 1**


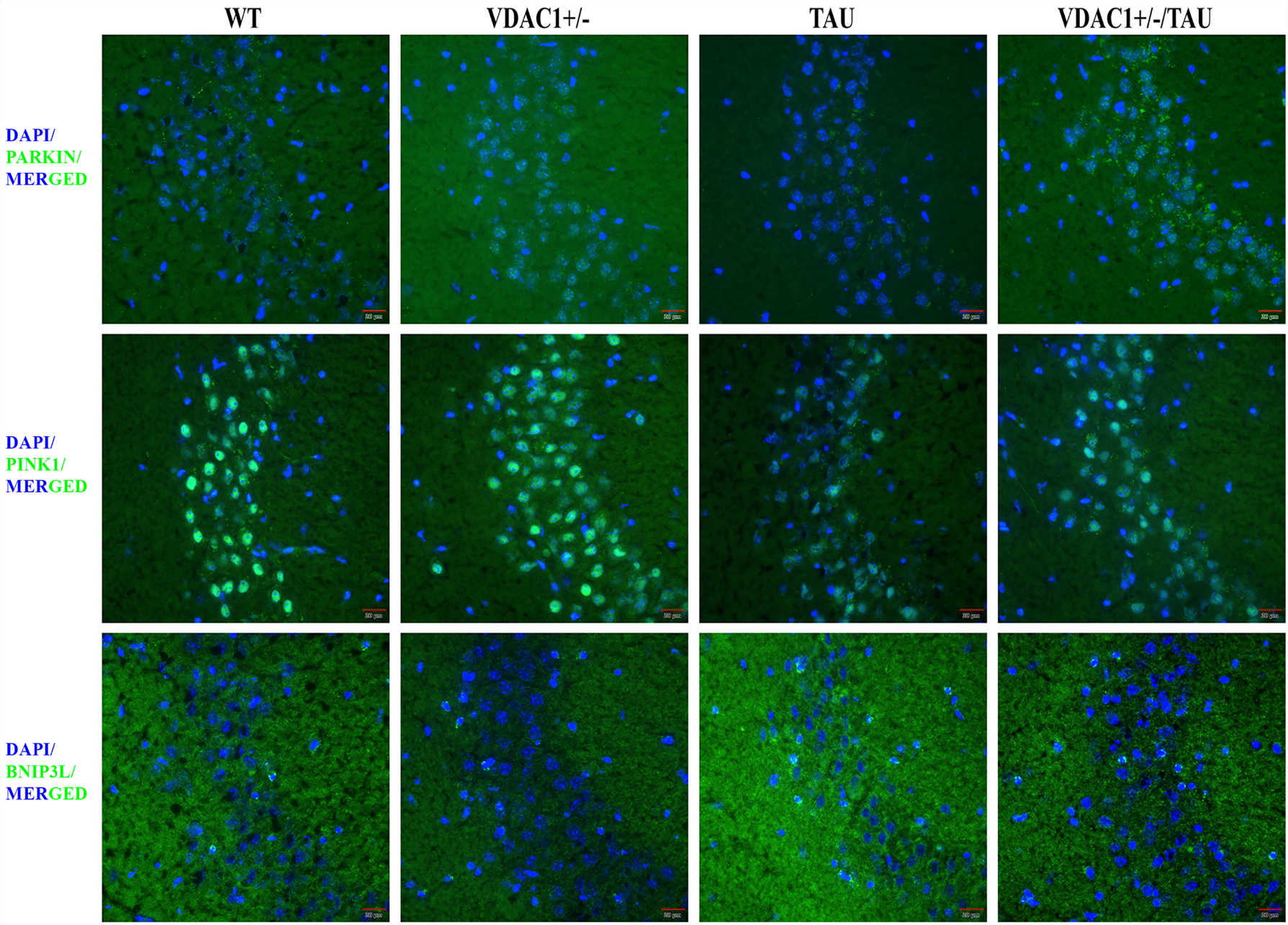


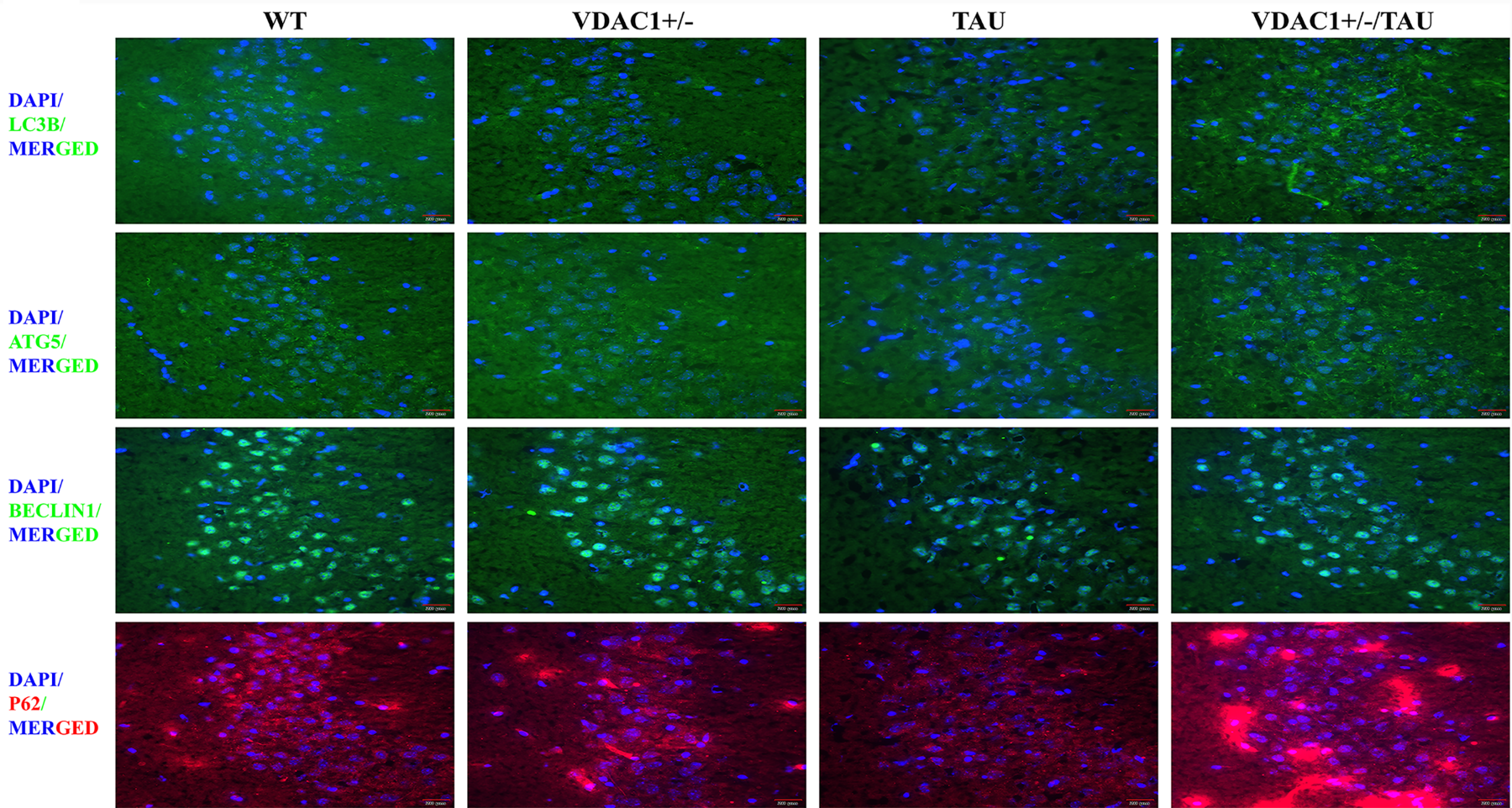


**Supplementary Figure 2**


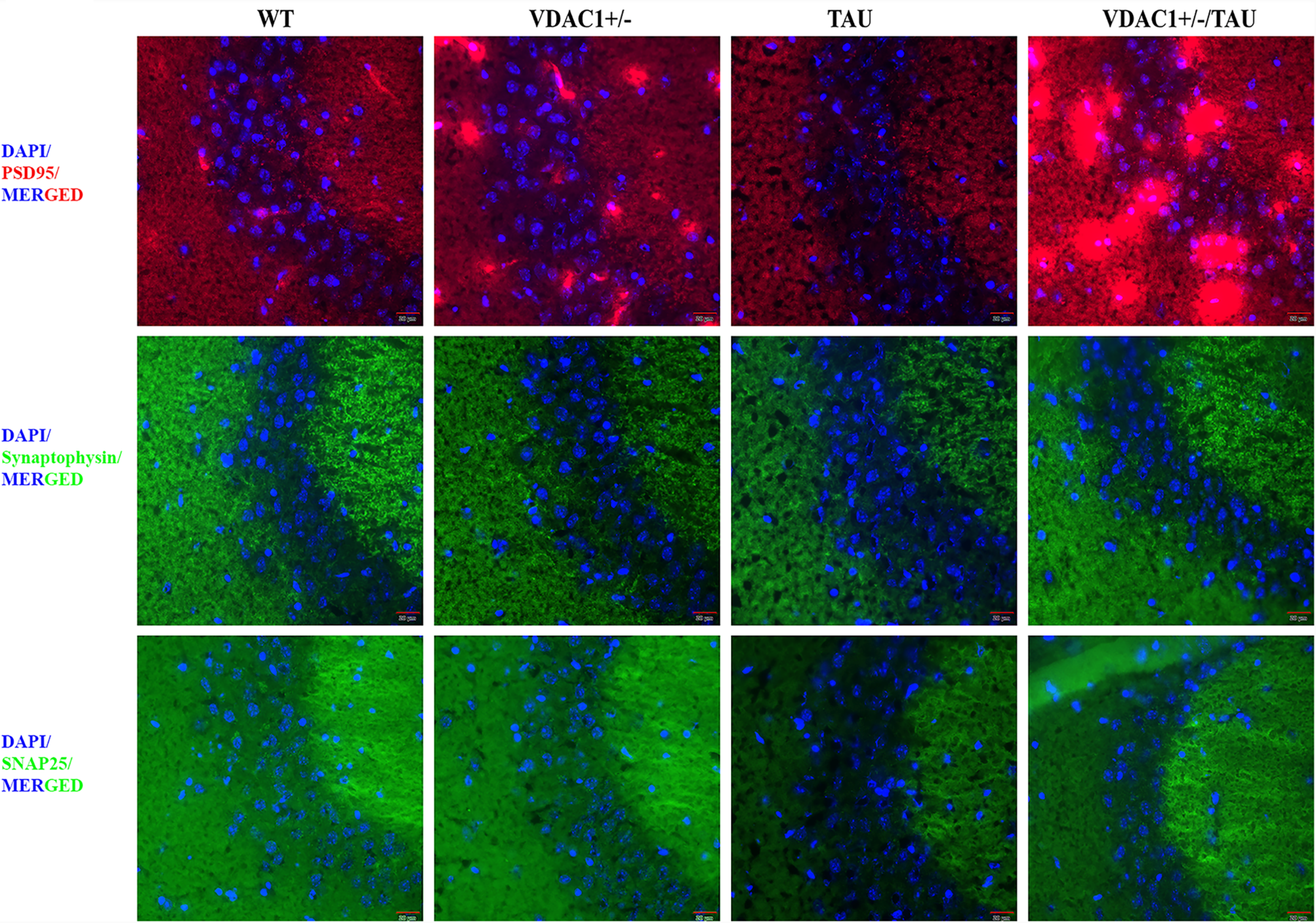


**Supplementary Figure 3**


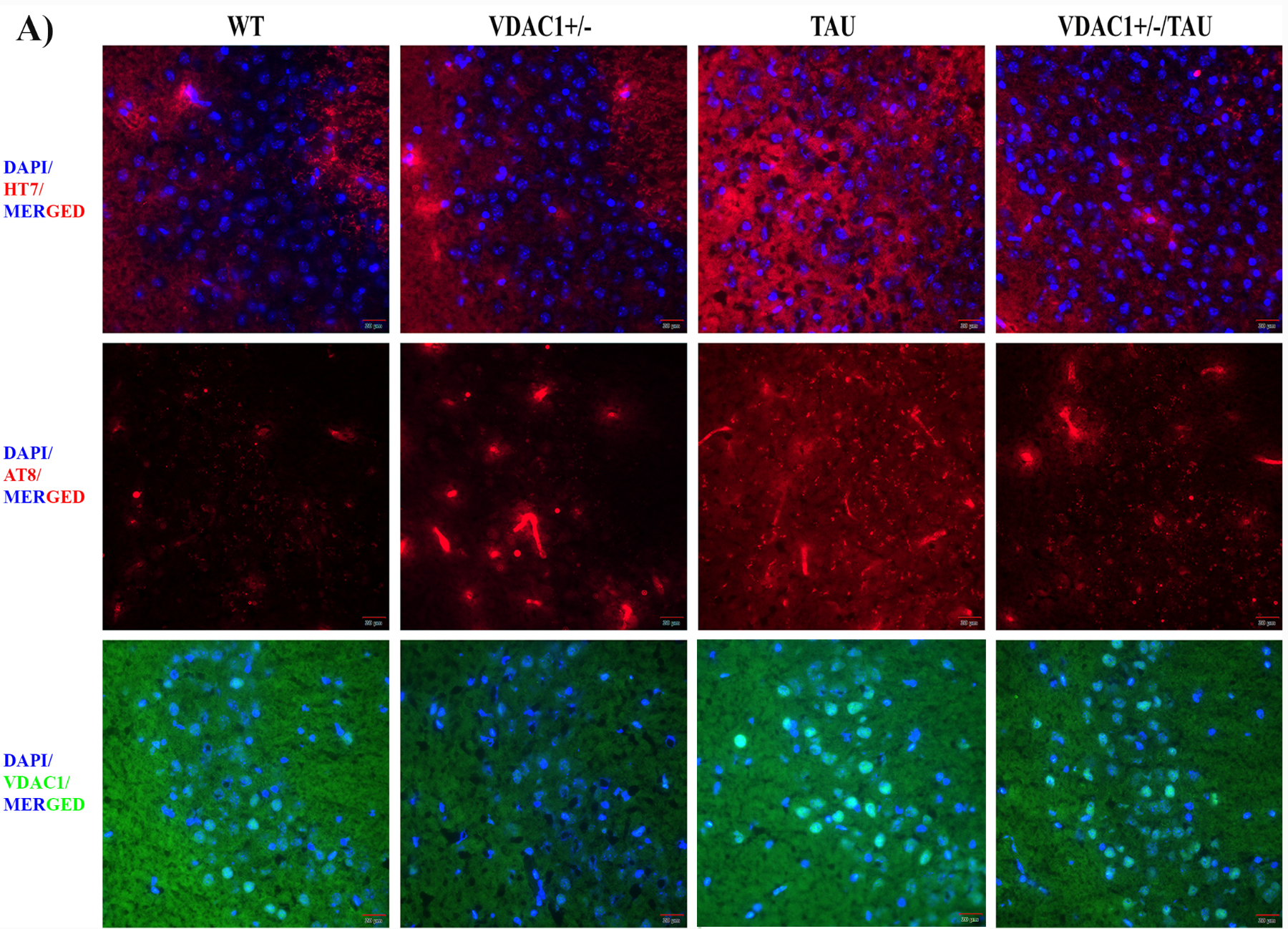


**Supplementary Figure 4A**


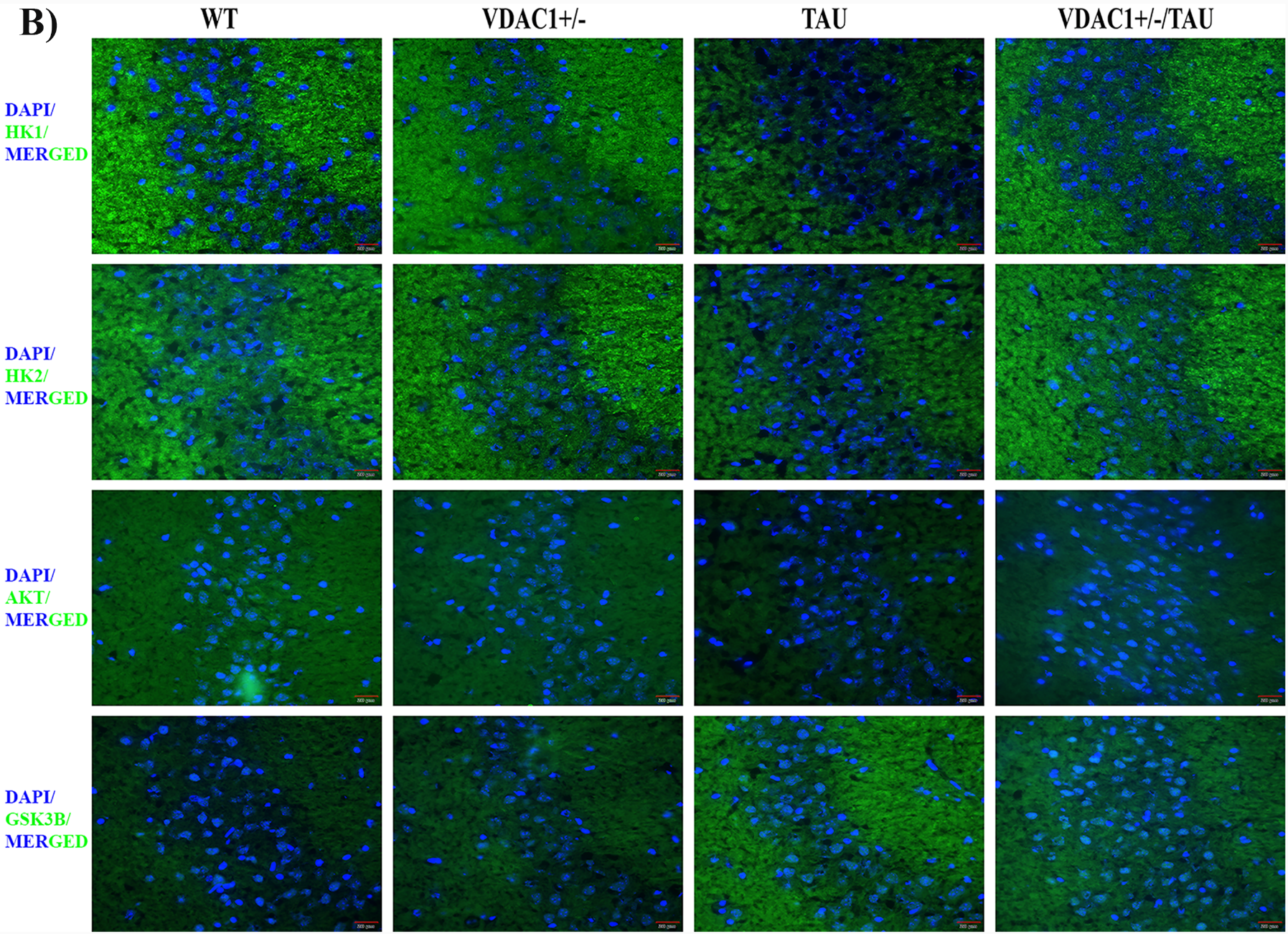


**Supplementary Figure 4B**


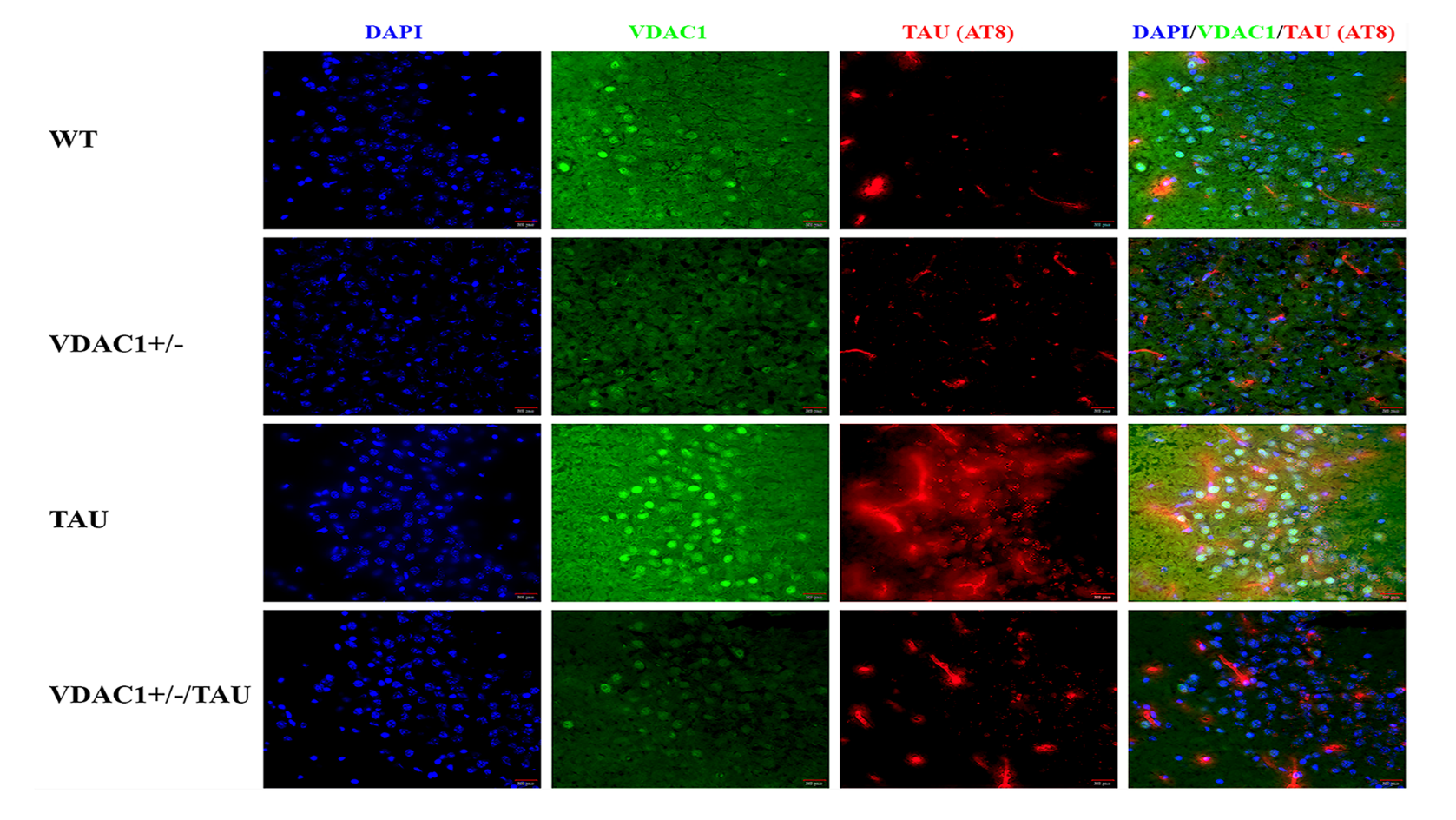


**Supplementary Figure 5**

**Supplementary Figure 6**


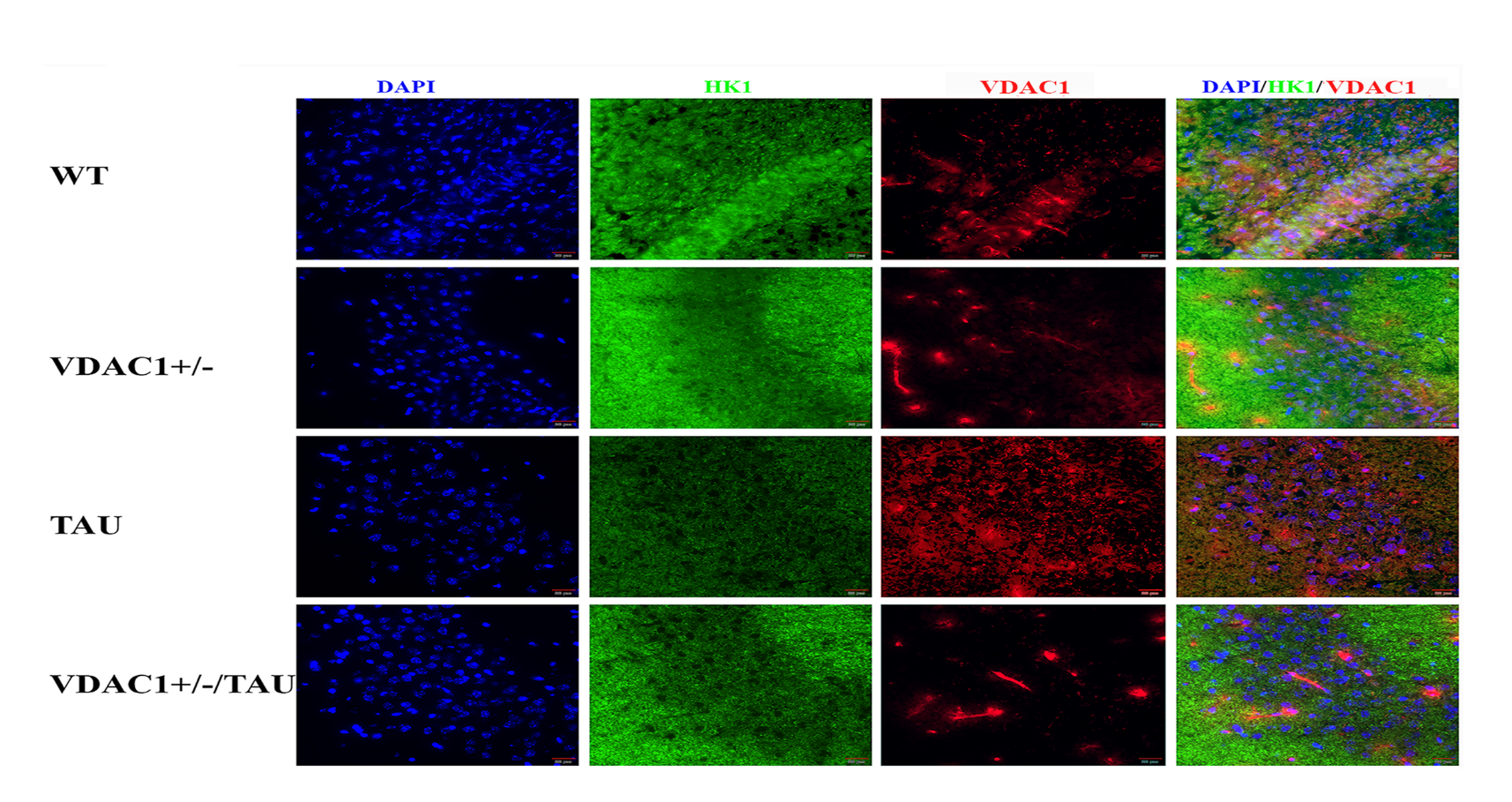


**Supplementary Figure 7**


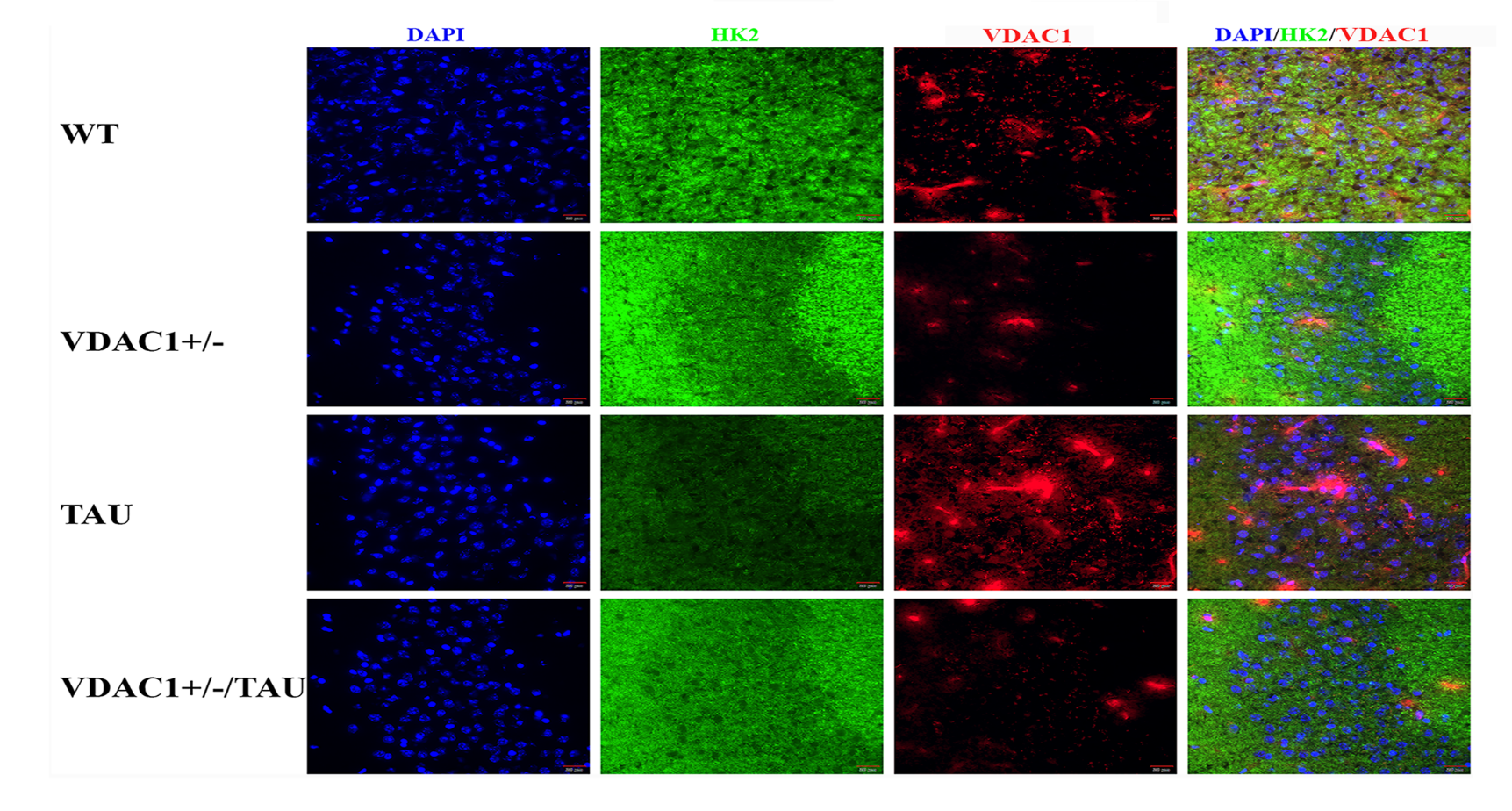


**Supplemental Figure Legends**

**Supplemental Figure 1.**

**Immunofluorescence images of mitophagy proteins in the hippocampal fields of 6-months-old WT, VDAC1^+/-^, TAU, and VDAC1^+/-^/TAU mice.** Representative Immunofluorescence images of 10 micron coronal sections (60X) of mitophagy proteins PARKIN, PINK1, BNIP3L. Data are from three independent experiments with similar results (N=3). Scale bar: 20 μm.

**Supplemental Figure 2.**

**Immunofluorescence images of autophagy proteins in the hippocampal fields of 6-months-old WT, VDAC1^+/-^, TAU, and VDAC1^+/-^/TAU mice.** Representative Immunofluorescence images of 10 micron coronal sections (60X) of autophagy proteins LC3B, ATG5, Beclin1, P62. Data are from three independent experiments with similar results (N=3). Scale bar: 20 μm.

**Supplemental Figure 3.**

**Immunofluorescence images of synaptic proteins in the hippocampal fields of 6-months-old WT, VDAC1^+/-^, TAU, and VDAC1^+/-^/TAU mice.** Representative Immunofluorescence images of 10 micron coronal sections (60X) of synaptic proteins PSD95, Synaptophysin, SNAP25. Data are from three independent experiments with similar results (N=3). Scale bar: 20 μm.

**Supplemental Figure 4.**

**Immunofluorescence images of other key proteins (Total TAU, P-TAU, VDAC1, HK1, HK2, AKT, GSK3B) in the hippocampal fields of 6-months-old WT, VDAC1^+/-^, TAU, and VDAC1^+/-^/TAU mice.** (A) Representative Immunofluorescence images of 10 micron coronal sections (60X) of other key proteins total TAU, P-TAU, VDAC1. (B) HK1, HK2, AKT, and GSK3B. Data are from three independent experiments with similar results (N=3). Scale bar: 20 μm.

**Supplemental Figure 5.**

**Double-labeling immunofluorescence analysis of VDAC1 and phosphorylated tau in the hippocampal fields of 6-months-old WT, VDAC1^+/-^, TAU, and VDAC1^+/-^/TAU mice.** Representative overview panel of coronal sections. The localization of VDAC1, phosphorylated tau (AT8) and the colocalization of VDAC1 and phosphorylated tau (merged) at 60X original magnification. Data are from three independent experiments with similar results (N=3). Scale bar: 20 μm.

**Supplemental Figure 6.**

**Double-labeling immunofluorescence analysis of HK1 and VDAC1 in the hippocampal fields of 6-months-old WT, VDAC1^+/-^, TAU, and VDAC1^+/-^/TAU mice.** Representative overview panel of coronal sections. The localization of HK1, VDAC1 and the colocalization of HK1 and VDAC1 (merged) at 60X original magnification. Data are from three independent experiments with similar results (N=3). Scale bar: 20 μm.

**Supplemental Figure 7.**

**Double-labeling immunofluorescence analysis of HK2 and VDAC1 in the hippocampal fields of 6-months-old WT, VDAC1^+/-^, TAU, and VDAC1^+/-^/TAU mice.** Representative overview panel of coronal sections. The localization of HK2, VDAC1 and the colocalization of HK2 and VDAC1 (merged) at 60X original magnification. Data are from three independent experiments with similar results (N=3). Scale bar: 20 μm.
