# Supplementary material for: A partial reduction of VDAC1 enhances mitophagy, autophagy, synaptic activities in a transgenic Tau mouse model
Source: Aging Cell. 2022 Jul 7;21(8):e13663. doi: 10.1111/acel.13663 (PMC9381918; doi:10.1111/acel.13663)
Supplement: Supplementary file 3 — Appendix S1 [file ACEL-21-e13663-s002.docx]

**Supplementary Material**

**Behavioral tests**

**Rotarod test**

A rotarod test was first used to measure the balance, coordination, and motor-planning of 6- month-old WT, VDAC1^+/-^, TAU (P301L), and VDAC1^+/-^/TAU mice. The mice were placed on the mouse rotarod (MedAssociates, Inc.) unit to test balance and motor coordination differences. Rotarod test behavioral assessment was performed as previously described. ^1^ The rod was set at 2 rpm on the apparatus, and mice were put, four at a time, each in an assigned position on the rod. Over a 5-min session, the rod was allowed to accelerate up to 40 rpm gradually. An IR beam is interrupted when the animal falls, halting the motor and the timer. We recorded the time spent on the rod without falling down, with a maximum of 5 minutes. Mice were subsequently removed and allowed to rest for 30 minutes before they returned to the remaining two test sessions, generating three trials per day over three consecutive days. The evaluation was done before the mouse fell down by tracking latency to fall and maximum rotation rate.

**Open field test**

Open Field was used to assay general locomotor activity levels, willingness to explore, and anxiety-related behavior and tracked using the ANY-Maze® software (Stoelting, Wood Dale, IL, USA). ^1^ In a 40 cm, square open field with video tracking tools, 6- month-old WT, VDAC1^+/-^, TAU (P301L), and VDAC1^+/-^/TAU mice were tracked for 5-min and under moderate lighting. The general activity levels were measured by assessing the total distance traveled, average speed, number of center entries, and time spent in the center. ^1^

**Y-maze test**

Spatial learning and memory have been shown to be sensitive to hippocampal damage. ^2^ Spontaneous alternation was determined by Y-maze tests for habituation and spatial working memory. ^1,2^ 6- month-old WT, VDAC1^+/-^, TAU (P301L), and VDAC1^+/-^/TAU were allowed to explore all three arms of the Y-maze freely, and spontaneous alternation was calculated. Each mouse was placed in one of the arms and allowed one five-minute trial of free exploration of the three arms in the maze. The number of total arm choices and sequence of arm choices was recorded (ANY-Maze®, Stoelting, Wood Dale, IL, USA). ^1,2^

**Morris Water Maze test**

Morris water maze (MWM) was used to test spatial long-term memory and learning. ^1,3^ The MWM test was conducted in a 120-cm diameter, 50-cm deep tank filled with opacified water (Utrecht Art Supplies, Cranbury, NJ) kept at 25 ± 0.5°C. A platform with a 9 cm diameter was submerged 1 cm underwater surface in a quadrant. The tank was imaginarily divided into four quadrants (compass locations: NE, NW, SW, and SE). The platform was placed in the NW quadrant and remained at the same position during the whole experiment. Briefly, every group of animals was trained for four days in MWM, with four trials per day, with 15 minutes intertrial intervals, so that one group of the animal was tested within 4 days/week. Each trial was set to run for one minute, but a trial ended once the animal was positioned on the platform for 3 seconds. If the animal did not find the platform within one minute, they were placed on the platform using the net for 3 seconds. Every trial animal was then dried with a towel and placed into a holding cage. Animals were video tracked using ANY-Maze® software (Stoelting, Wood Dale, IL, USA), and behavioral parameters (average time to find the platform, distance traveled, average speed, and the number of entries in the NW quadrant) were automatically calculated. ^1,3^

**Immunoblotting analysis**

Cerebral cortex tissues were homogenized by sonication in RIPA buffer (Thermo Scientific, Catalog number: 89901) with Halt™ Protease and Phosphatase Inhibitor (Thermo Scientific: 78444), and EDTA. Protein lysates were initially incubated on ice for 20 min, and lysates were thoroughly resuspended and incubated for 20 minutes on ice with the occasional swirling of tubes. Further lysates were clarified by centrifugation at 4°C for 20 min at 13,000 × g. The protein concentrations were determined by BCA protein assay (Thermo Scientific: 23222). Homogenate was stored at −80°C. Equal amounts of protein were diluted in 4X Bolt™ LDS Sample Buffer (Thermo Scientific: B0007) and electrophoresed in 10%, 12%, and 4-20% Mini-PROTEAN® TGX Precast Protein Gels (BIO-RAD). The proteins were then transferred to PVDF membranes using Trans-Blot Turbo Transfer System (BIO-RAD). The membranes were blocked using 5% Bovine Serum Albumin (BSA) or 5% nonfat skimmed milk for 1 h. Immunoblotting analysis was performed for mitophagy, autophagy, mitochondrial dynamics, biogenesis, synaptic, and other key proteins. Details of antibody dilutions were given in Supplemental Table 1. The membranes were washed with a TBST buffer three times at 10-min intervals and were then incubated for 1h with appropriate secondary antibodies, followed by three additional washes at 10-min intervals. The blots were detected in chemiluminescent detection using ECL with ImageQuant LAS-4000 (GE Healthcare Life Sciences). The intensity of the various protein bands was quantified using ImageJ software.

**Immunofluorescence**

Mouse brain sections were prepared for immunofluorescence as previously described. ^4^ Coronal sections were used for immunofluorescence. Sections were first fixed with 1% paraformaldehyde in PBS for 10 min and then washed three times with TBST. Then the sections were blocked with SuperBlock™ (PBS) Blocking Buffer (Thermo Scientific: 37515) at room temperature for one hour. Sections were then incubated with the respective primary antibodies (Supplemental Table 2) overnight at 4°C. After incubation, the tissues were washed three times with TBST for 10 min each. The tissues were then ready to be incubated with a secondary antibody conjugated with Alexa Fluor 488-goat-anti-rabbit (Invitrogen: A32731), Alexa Flour 594-goat-anti-mouse (Invitrogen: A11005), at room temperature for one hour. The slides were exposed to DAPI nuclear staining for 15 min before being sealed with antifade mounting medium and glass covers. Immunofluorescent sections were visualized, and images were captured using an Olympus IX83 microscope (Olympus, USA). ImageJ software measured the quantification of staining intensity and background fluorescence intensity. The intensity of specific staining was calculated as previously described. ^4^ Quantification of the colocalization of VDAC1 and p-TAU, HK1 and VDAC1, HK2 and VDAC1 was conducted using ImageJ software*.* Photographs were taken of 10-15 fields of each section at 10X, and 60X magnifications; the relative immunoreactivities of these antibodies were quantified, and statistical significance was assessed. A quantitative analysis using NIH ImageJ was performed on Background-subtracted digital images. ^4^

**References**

1. Vijayan M, Bose C, Reddy PH. Protective effects of a small molecule inhibitor, DDQ against amyloid beta in Alzheimer's disease. *Mitochondrion*. Jul 2021;59:17-29. doi:10.1016/j.mito.2021.04.005

2. Hegde V, Vijayan M, Kumar S, et al. Adenovirus 36 improves glycemic control and markers of Alzheimer's disease pathogenesis. *Biochim Biophys Acta Mol Basis Dis*. Nov 1 2019;1865(11):165531. doi:10.1016/j.bbadis.2019.08.007

3. Vijayan M, Bose C, Reddy PH. Anti-brain Aging Effects of Small Molecule Inhibitor DDQ. *Mol Neurobiol*. Jul 2021;58(7):3588-3600. doi:10.1007/s12035-021-02360-7

4. Vijayan M, George M, Bunquin LE, Bose C, Reddy PH. Protective effects of a small-molecule inhibitor DDQ against tau-induced toxicities in a transgenic tau mouse model of Alzheimer's disease. *Hum Mol Genet*. Sep 24 2021;doi:10.1093/hmg/ddab285
